# Triple therapy with artemether–lumefantrine plus amodiaquine versus artemether–lumefantrine alone for artemisinin-resistant, uncomplicated falciparum malaria: an open-label, randomised, multicentre trial

**DOI:** 10.1016/S1473-3099(21)00692-7

**Published:** 2022-06

**Authors:** Thomas J Peto, Rupam Tripura, James J Callery, Dysoley Lek, Ho Dang Trung Nghia, Chea Nguon, Nguyen Thi Huyen Thuong, Rob W van der Pluijm, Nguyen Thi Phuong Dung, Meas Sokha, Vo Van Luong, Le Thanh Long, Yok Sovann, Jureeporn Duanguppama, Naomi Waithira, Richard M Hoglund, Palang Chotsiri, Nguyen Hoang Chau, Andrea Ruecker, Chanaki Amaratunga, Mehul Dhorda, Olivo Miotto, Richard J Maude, Huy Rekol, Kesinee Chotivanich, Joel Tarning, Lorenz von Seidlein, Mallika Imwong, Mavuto Mukaka, Nicholas P J Day, Tran Tinh Hien, Nicholas J White, Arjen M Dondorp

**Affiliations:** a Mahidol Oxford Tropical Medicine Research Unit, Faculty of Tropical Medicine, Mahidol University, Bangkok, Thailand; b Centre for Tropical Medicine and Global Health, Nuffield Department of Clinical Medicine, University of Oxford, Oxford, UK; c National Center for Parasitology, Entomology and Malaria Control, Phnom Penh, Cambodia; d School of Public Health, National Institute of Public Health, Phnom Penh, Cambodia; e Oxford University Clinical Research Unit, Hospital for Tropical Diseases, Ho Chi Minh City, Vietnam; f Pham Ngoc Thach University of Medicine, Ho Chi Minh City, Vietnam; g Pailin Provincial Health Department, Pailin, Cambodia; h Faculty of Tropical Medicine, Mahidol University, Bangkok, Thailand; i WorldWide Antimalarial Resistance Network, Asia-Pacific Regional Centre, Bangkok, Thailand; j Wellcome Trust Sanger Institute, Hinxton, UK; k Harvard T H Chan School of Public Health, Harvard University, Boston, MA, USA; l The Open University, Milton Keynes, UK; m Department of Clinical Tropical Medicine, Mahidol University, Bangkok, Thailand; n Department of Molecular Tropical Medicine and Genetics, Faculty of Tropical Medicine, Mahidol University, Bangkok, Thailand

## Abstract

**Background:**

Late treatment failures after artemisinin-based combination therapies (ACTs) for falciparum malaria have increased in the Greater Mekong subregion in southeast Asia. Addition of amodiaquine to artemether–lumefantrine could provide an efficacious treatment for multidrug-resistant infections.

**Methods:**

We conducted an open-label, randomised trial at five hospitals or health centres in three locations (western Cambodia, eastern Cambodia, and Vietnam). Eligible participants were male and female patients aged 2–65 years with uncomplicated *Plasmodium falciparum* malaria. Patients were randomly allocated (1:1 in blocks of eight to 12) to either artemether–lumefantrine alone (dosed according to WHO guidelines) or artemether–lumefantrine plus amodiaquine (10 mg base per kg/day), both given orally as six doses over 3 days. All received a single dose of primaquine (0·25 mg/kg) 24 h after the start of study treatment to limit transmission of the parasite. Parasites were genotyped, identifying artemisinin resistance. The primary outcome was Kaplan-Meier 42-day PCR-corrected efficacy against recrudescence of the original parasite, assessed by intent-to-treat. Safety was a secondary outcome. This completed trial is registered at ClinicalTrials.gov (NCT03355664).

**Findings:**

Between March 18, 2018, and Jan 30, 2020, 310 patients received randomly allocated treatment; 154 received artemether–lumefantrine alone and 156 received artemether–lumefantrine plus amodiaquine. Parasites from 305 of these patients were genotyped. 42-day PCR-corrected treatment efficacy was noted in 151 (97%, 95% CI 92–99) of 156 patients with artemether–lumefantrine plus amodiaquine versus 146 (95%, 89–97) of 154 patients with artemether–lumefantrine alone; hazard ratio (HR) for recrudescence 0·6 (95% CI 0·2–1·9, p=0·38). Of the 13 recrudescences, 12 were in 174 (57%) of 305 infections with *pfkelch13* mutations indicating artemisinin resistance, for which 42-day efficacy was noted in 89 (96%) of 93 infections with artemether–lumefantrine plus amodiaquine versus 73 (90%) of 81 infections with artemether–lumefantrine alone; HR for recrudescence 0·44 (95% CI 0·14–1·40, p=0·17). Artemether–lumefantrine plus amodiaquine was generally well tolerated, but the number of mild (grade 1–2) adverse events, mainly gastrointestinal, was greater in this group compared with artemether–lumefantrine alone (vomiting, 12 [8%] with artemether–lumefantrine plus amodiaquine *vs* three [2%] with artemether–lumefantrine alone, p=0·03; and nausea, 11 [7%] with artemether–lumefantrine plus amodiaquine *vs* three [2%] with artemether–lumefantrine alone, p=0·05). Early vomiting within 1 h of treatment, requiring retreatment, occurred in no patients of 154 with artemether–lumefantrine alone versus five (3%) of 156 with artemether–lumefantrine plus amodiaquine, p=0·06. Bradycardia (≤54 beats/min) of any grade was noted in 59 (38%) of 154 patients with artemether–lumefantrine alone and 95 (61%) of 156 with artemether–lumefantrine plus amodiaquine, p=0·0001.

**Interpretation:**

Artemether–lumefantrine plus amodiaquine provides an alternative to artemether–lumefantrine alone as first-line treatment for multidrug-resistant *P falciparum* malaria in the Greater Mekong subregion, and could prolong the therapeutic lifetime of artemether–lumefantrine in malaria-endemic populations.

**Funding:**

Bill & Melinda Gates Foundation, Wellcome Trust.

## Introduction

The countries of the Greater Mekong subregion in southeast Asia are committed to eliminate *Plasmodium falciparum* malaria by 2030.^1^ A strong incentive for rapid elimination is the increasing problem of multidrug-resistant *P falciparum* in the region and the risk of this spreading globally.^2^ Uncomplicated falciparum malaria is treated with artemisinin-based combination therapies (ACTs), and there are currently no adequate alternatives. New antimalarial compounds are not expected to come to market before 2027.^3^

**Research in context**

**Evidence before this study**

Artemisinin-based combination therapies (ACTs) are the first-line treatment for falciparum malaria, but parasites that are resistant to artemisinin and to the partner drugs used in ACTs have emerged, with high levels of resistance noted in Greater Mekong subregion countries such as Cambodia and Vietnam. Resistance to artemisinin and ACT partner drugs has been reported widely from Cambodia and Vietnam since 2009, with a single artemisinin-resistant parasite lineage carrying the *C580Y pfkelch13* mutation becoming the predominant artemisinin-resistant strain by 2016–17. To improve cure rates and extend the useful therapeutic lifetimes of the available drugs, artemisinin could be given with two partner drugs, lumefantrine and amodiaquine, as triple ACT. We searched PubMed for articles published before Aug 22, 2021, using the terms “falciparum malaria”, “amodiaquine”, and “lumefantrine”, in combination with either “Cambodia” or “Vietnam”. Of 44 articles found, one reported the efficacy of artemether–lumefantrine plus amodiaquine for the treatment of uncomplicated falciparum malaria mainly in Asian countries without ACT treatment failure and was not conducted in Cambodia or Vietnam. In this large randomised controlled trial, adding amodiaquine to artemether–lumefantrine was well tolerated (although it slightly increased the incidence of vomiting and mild bradycardia), and effective. Pharmacokinetic analysis suggested that the addition of amodiaquine to artemether–lumefantrine might reduce exposure to lumefantrine. The efficacy of artemether–lumefantrine alone for the treatment of uncomplicated falciparum malaria was reported in two small Cambodian studies covering the period 2003–11. The PCR-corrected 28-day efficacy against recrudescence was 71% when artemether–lumefantrine was given without the fatty food needed to facilitate lumefantrine absorption but was 82% to 87% when given with fatty food. In a Cambodian study covering the period 2016–17, the 28-day efficacy of artesunate–amodiaquine was 81%.

**Added value of this study**

This is the first randomised trial of artemether–lumefantrine alone versus artemether–lumefantrine plus amodiaquine for uncomplicated falciparum malaria in areas with a high prevalence of artemisinin resistance. It showed that although artemether–lumefantrine alone achieves apparently complete clearance of artemisinin-resistant infections, it had only about 90% efficacy against recrudescence; for such infections, triple therapy with artemether–lumefantrine plus amodiaquine is a safe option that might be about 96% effective against recrudescence, although this apparent gain in efficacy was not statistically significant. The addition of amodiaquine to artemether–lumefantrine did not affect plasma lumefantrine concentrations but, as in a previous trial, it did slightly increase the incidence of vomiting and of mild bradycardia.

**Implications of all the available evidence**

The triple ACT artemether–lumefantrine plus amodiaquine is a well tolerated and effective treatment for uncomplicated falciparum malaria in an area with multidrug-resistant parasites. If, as the present study suggests, the addition of amodiaquine to artemether–lumefantrine approximately halves the risk of recrudescence could be the preferred treatment in areas with artemisinin resistance. Artemether–lumefantrine plus amodiaquine provides an alternative first-line treatment for uncomplicated falciparum malaria in southeast Asia and elsewhere, with an expected longer useful therapeutic lifetime than currently used ACTs.

Artemisinin resistance was first reported in 2009 from western Cambodia, and has since spread throughout the Greater Mekong subregion.^2^, ^4^, ^5^ This resistance has been compounded by ACT partner drug resistance, and all of the current six recommended ACTs have shown reduced efficacy in some Greater Mekong subregion countries at some point.^6^ The availability of effective treatment is a prerequisite for malaria elimination. The strategy in the Greater Mekong subregion has been to switch to an alternative ACT when the efficacy of the first-line treatment falls below 90%, following WHO recommendations. Cambodia started using artesunate–mefloquine in 2000, then changed its first-line treatment to dihydroartemisinin–piperaquine in 2010, then gradually changed back to artesunate–mefloquine during 2014 to 2017. Vietnam began using mefloquine as monotherapy or as artesunate–mefloquine in the 1990s, changed its first-line treatment to dihydroartemisinin–piperaquine in 2005, and has used artesunate–pyronaridine in four provinces since 2020.

An alternative to the programmatically difficult rotation between ACTs is the use of triple ACTs, chosen to combine the short-acting artemisinin component with two longer-acting partner drugs matched for their pharmacokinetic profile but differing in their parasite-resistance profiles.^6^ The use of triple ACTs could extend the useful therapeutic lifetimes of existing antimalarials, because of the mutual protection between the two partner drugs. Artemether–lumefantrine combined with amodiaquine is a promising triple-drug combination, as the pharmacokinetic profiles of lumefantrine and desethyl-amodiaquine (the active amodiaquine metabolite) are well matched and the two drugs have different and potentially opposing resistance mechanisms.^7^, ^8^

A previous study evaluated the efficacy of artemether–lumefantrine plus amodiaquine in regions with a low prevalence of artemisinin resistance and partner drug resistance (Democratic Republic of the Congo, India, Bangladesh, Myanmar, and Laos),^9^ but information is scarce regarding the efficacy of artemether–lumefantrine plus amodiaquine in countries with high levels of resistance such as Cambodia and Vietnam. Additionally, the efficacy of artemether–lumefantrine alone has not been evaluated in these countries since 2005.^10^, ^11^ We conducted an open-label randomised controlled trial in Cambodia and Vietnam comparing efficacy, safety, and tolerability of artemether–lumefantrine alone versus artemether–lumefantrine for the treatment of uncomplicated falciparum malaria.

## Methods

### Study design and participants

We conducted an open-label, randomised trial in hospitals or health centres at five sites in three locations: western Cambodia (Pursat and Pailin), eastern Cambodia (Stung Treng), and Vietnam (Binh Phuoc and Khanh Hoa). The trial was approved by the National Ethics Committee for Health Research (NECHR; Phnom Penh, Cambodia); the Ethical Committee, Hospital for Tropical Diseases (Ho Chi Minh City, Vietnam; and Oxford University Tropical Research Ethics Committee (OXTREC; Oxford, UK). Oxford University was the study sponsor. Monitoring was done by the Mahidol-Oxford Tropical Medicine Research Unit (MORU; Bangkok, Thailand) and the Oxford University Clinical Research Unit (Ho Chi Minh City, Vietnam). Ethics approval was obtained from the NECHR, Cambodia (NECHR 0042); the Ethical Committee, Hospital for Tropical Diseases, Vietnam (1096); and OXTREC (32–17).

Male and female patients aged 2–65 years were eligible to participate if they had uncomplicated *P falciparum* malaria (or mixed *P falciparum* plus another *Plasmodium* sp) with microscopically confirmed *P falciparum* parasitaemia less than 200 000 parasites per μL, and either fever of 37·5°C or more or a history of fever within the previous 24 h. Exclusion criteria were severe malaria, another acute illness requiring treatment, a prolonged Bazett-corrected QT-interval of more than 450 ms on an electrocardiogram (ECG), pregnancy, breastfeeding, use of artemisinin-based treatment in the previous 7 days, a history of splenectomy, an allergy to study drugs, a haematocrit concentration of less than 25%, or participation in any trial in the past 3 months. For the recruitment process, patients identified in villages by local health workers were referred to a nearby study centre, to which those who entered the trial were admitted for the duration of their treatment. Informed consent was obtained in writing from patients or a parent, guardian, or witness. Additionally, informed assent was obtained from patients aged 12–18 years.

### Randomisation and masking

Eligible patients were randomly assigned (1:1, in blocks of size eight to 12 unknown to study staff) at each study site to either artemether–lumefantrine alone or artemether–lumefantrine plus amodiaquine. The sequence was computer-generated by the trial statistician who also supervised preparation of the randomisation envelopes. Allocation was done by opening the next-numbered opaque envelope containing the study number and treatment. Once that envelope was opened, the patient was included in the study. Although this was an open-label trial, to minimise potential bias the laboratory assessments, centrally and at the study sites, were all performed blindly.

### Procedures

Patients were admitted into the study ward and given either artemether–lumefantrine alone or artemether–lumefantrine plus amodiaquine, both administered orally as six doses over 3 days and directly observed by a member of the study team. Drugs were given with a fatty snack; eg, 80 mL of milk. Artemether–lumefantrine was dosed according to WHO guidelines (appendix p 7). The target dose of amodiaquine was 10 mg base per kg/day, given as a split dose twice daily (together with artemether–lumefantrine). Patients also received a single gametocytocidal dose of primaquine (0·25 mg/kg) 24 h after the start of study treatment to limit transmission of the parasite. To determine asexual parasite clearance half-life, Giemsa-stained blood smears were taken at 6-h intervals until clearance was apparent by microscopy on two consecutive smears.^12^ Patients remained in the study ward until parasite clearance and completion of treatment.

After discharge, patients were assessed weekly as outpatients from week 1 to week 6 after study entry, including a peripheral blood smear for immediate microscopy. Antimalarial treatment was provided if any recurrent *Plasmodium* spp infection was detected. At day 0, day 7, and whenever there was a recurrence during follow-up, blood was taken into EDTA (edetic acid) vacutainers and into heparinised vacutainers, separated, stored locally either at −80°C or over liquid nitrogen, and transported on dry ice for storage and assay in Bangkok (Thailand).

Following WHO guidelines,^13^ recurrent infections during the 6-week follow-up were classified as recrudescent if genotyping showed that *P falciparum msp1, msp2*, and *glurp* alleles matched those present at baseline. Safety monitoring included full blood counts and blood biochemistry at baseline, day 3, day 7, and day 28; ECGs just before and 4 hours after each treatment dose, and on day 28; and physical examinations and systematic symptom questionnaires every 24 h in the study ward and at each follow-up visit. Adverse events were graded according to the Division of AIDS 2017 criteria.^14^ In a subset of Vietnamese patients, frequent blood sampling was conducted for more detailed pharmacokinetic analyses of study drugs and their metabolites (appendix, pp 8–10, 16–22).

For assessment of mutations in *P falciparum kelch13, Pfcrt, pfplasmepsin2*, and *Pfmdr1,* DNA was extracted from dried blood spots on filter paper. Nested PCR was performed to amplify the propeller region of the *pfkelch13* gene following published methods,^15^ then *pfkelch13* was genotyped by direct sequencing of the PCR product (Macrogen, Seoul, South Korea) and compared with National Center for Biotechnology Information reference sequence 3D7 (XM001350122.1; PF13_0238) using Bioedit.^15^ *Pfcrt* was amplified from the DNA template by nested PCR. A PCR-restriction-fragment-length polymorphism assay was used to assess mutations, including those related to piperaquine resistance (*N88K, T93S, H97Y, F145I, I218F, CVMNK72-76CVIET, N326S, M343L, G353V, I356T,* and *R371I*).^16^ For quality control, a third of all PCR products were randomly assessed by full DNA sequencing (Macrogen). *Pfplasmepsin2* and *pfmdr1* copy number variations were assessed with relative quantitative real-time PCR, using TaqmanTM on Corbett Rotor-Gene Q (Corbett Research, Sydney, Australia). Amplification was performed in triplicate by Quantitec Multiplex PCR no ROX (QIAGEN, Hilden, Germany), with standard primers and probes.^17^

*P falciparum* isolates from Cambodia had in-vitro lumefantrine susceptibility assessed.^18^, ^19^ Venous blood samples, cryopreserved with glycerolyte, were transported on dry ice to the MORU malaria laboratory in Bangkok. Thawed parasites were cultured for 4 h, then resuspended to 5% haematocrit in RPMI 1640, with 25 mM HEPES and 25 mM NaHCO_3_ supplemented with 10% human AB serum. Susceptibility was assessed by a 48-h in-vitro schizont maturation inhibition assay.^20^ For this, the cell suspension was seeded into a 96-well drug-precoated plate (range 0·2–1000·0 ng/mL lumefantrine) in duplicate, and incubated under 5% CO_2_ at 37°C until 20% of parasites in the drug-free control well reached the schizont stage, after which the number of schizont-infected erythrocytes (containing >8 merozoites) per 100 infected erythrocytes were counted with bright-field microscopy.

### Outcomes

For the randomised comparison of artemether–lumefantrine alone versus artemether–lumefantrine plus amodiaquine, the primary outcome was 42-day PCR-corrected efficacy against recrudescence. This is reported separately by study location, and for patients with and without artemisinin-resistant infections.

Secondary outcomes were safety, tolerability, vomiting, bradycardia, QT-interval prolongation, other adverse events, fever, and parasite clearance times (both stratified by *pfkelch13* mutation status), and the effects of amodiaquine on artemether–lumefantrine pharmacokinetics. Secondary outcomes not involving the randomised comparison (eg, parasite genomics, transcriptomics, gametocyte dynamics, and in-vitro drug sensitivity) will be reported separately.

### Statistical analysis

This report follows the pre-specified statistical analysis plan. The original power calculations for the primary outcome assumed that most patients in the study areas would have artemisinin-resistant infections and that the addition of amodiaquine to artemether–lumefantrine would increase efficacy from 90% to 99%. To have an 80% chance of detecting this at each site with a statistical significance level of 0·05, a sample size of 100 patients per group at each of the three locations would be required. The main analyses of the primary outcome were by intent-to-treat (ITT), pooled across sites, and used Kaplan-Meier estimates. As follow-up was to be weekly for six visits, time to any recurrent infection during follow-up was rounded to the nearest whole number of weeks. Patients who were not seen for the visit at week 6 were censored at the week of last follow-up. Participants who had a reinfection during follow-up with a new *P falciparum* strain, or with *Plasmodium vivax*, were censored when it was discovered (as treatment of the reinfection could prevent recrudescence of the original infection). We obtained day-42 recrudescence-free survival estimates using interval-censored Kaplan-Meier analysis. We used unpaired *t* tests to compare changes in heart rate, changes in Bazett-corrected QTc-interval, and parasite clearance half-lives between treatment groups. Proportions were compared with Fisher's exact test. Analyses used Stata version 16.

Following standard methods, copy number estimates of mutations in *pfkelch13, Pfcrt, pfplasmepsin2*, and *pfmdr1* were calculated as 2^−∆∆Ct^ (with ∆∆ Ct denoting the difference between ∆ Ct of the unknown sample and ∆ Ct of the reference sample). Reactions were repeated if the SD of ∆∆ Ct was more than 1·5, the Ct was more than 35, or the profile did not conform to exponential kinetics. The half-maximal inhibitory concentration (IC_50_) for in-vitro lumefantrine susceptibility was determined by nonlinear regression of the log dose-response curves (GraphPad Prism version 8.2.1). *P falciparum* Thai laboratory strain TM267 was used as a reference for quality control of the assay with a mean lumefantrine IC_50_ of 19 (SD 9) ng/mL. The cutoff value for reduced susceptibility was 80 ng/mL.

For drug quantification and pharmacokinetics, lumefantrine and desbutyl-lumefantrine concentrations were assessed at day 7 in all patients with median plasma concentrations and IQRs reported. Additionally, some Vietnamese participants were sampled frequently for detailed assessment of plasma concentrations of lumefantrine and desbutyl-lumefantrine; artemether and dihydroartemisinin; and amodiaquine and desethyl-amodiaquine. Drug quantification assays used liquid chromatography with tandem mass spectrometry.^21^, ^22^, ^23^ Pharmacokinetic parameters were obtained from non-compartmental pharmacokinetic analyses of the dense plasma data, using PKanalix v2019R1 (Antony, France: Lixoft SAS, 2019). Statistical and graphical analyses were performed using R version 3.6.0 and GraphPad Prism. A data and safety monitoring board met before the trial and at three points during recruitment. The trial is registered with ClinicalTrials.gov, NCT03355664, and is now complete.

### Role of the funding source

The funders of the study had no role in study design, data collection, data analysis, data interpretation, or writing of the report.

## Results

**Figure 1:**
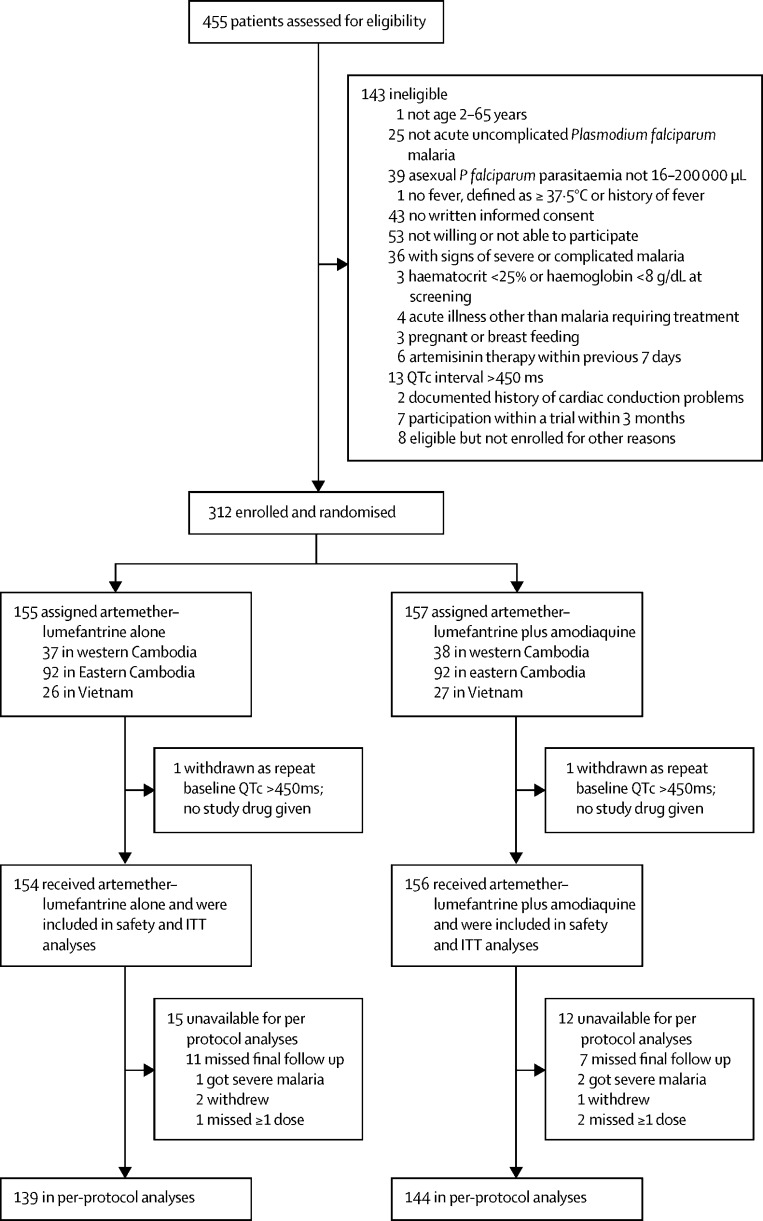
Trial profile ITT=intention-to-treat

Between March 18, 2018, and Jan 30, 2020, 455 patients were assessed for eligibility and 312 were enrolled and randomly assigned to treatment (figure 1), fewer than the 600 intended because of decreases in the incidence of falciparum malaria in the study areas. One patient in each treatment group was excluded because of a prolonged QTc interval on the ECG before treatment, with no study drug given to either patient; both recovered with standard treatment. Thus, 310 patients received study drugs, 154 received artemether–lumefantrine alone, and 156 received artemether–lumefantrine plus amodiaquine. A subset of 38 Vietnamese patients (20 assigned artemether–lumefantrine alone, 18 assigned artemether–lumefantrine plus amodiaquine) had frequent blood sampling conducted for more detailed pharmacokinetic analyses.

Baseline characteristics were generally well balanced (table 1). Participants were mostly male (88%), 53% had a fever, the median age was 25 years (IQR 18–34, range 4–58), and mean haemoglobin concentration was 12·7 g/dL (SD 1·7).

**Table 1:** Baseline characteristics of the intention-to-treat population

		**Artemether–lumefantrine alone (n=154)**	**Artemether–lumefantrine plus amodiaquine (n=156)**
Age, years		24 (18–34)	25 (19–33)
Sex			
	Male	142 (92%)	132 (85%)
	Female	12 (8%)	24 (15%)
Tympanic temperature ≥37·5°C		81 (53%)	83 (53%)
Weight, kg		51 (11)	51 (12)
Height, cm		158 (12)	157 (14)
Heart rate, bpm		89 (78–97)	90 (81–104)
Respiratory rate per min		25 (23–28)	26 (22–28)
Systolic blood pressure, mmHg		113 (11)	113 (12)
Diastolic blood pressure, mmHg		69 (8)	68 (8)
QTcB interval, ms		413 (18)	415 (17)
Haemoglobin, g/dL		12·8 (1·7)	12·7 (1·8)
*Pfkelch13* mutant*		81 (53%)	93 (60%)
*Pfkelch13* wild-type		69 (45%)	62 (40%)
*Plasmodium falciparum*, not genotyped		4 (3%)	1 (<1%)
Co-infection with *Plasmodium vivax*		22 (14%)	29 (19%)
Parasite count per μL, geometric mean		7670 (95% CI 5356–10 983)	11 647 (95% CI 8538–15 890)
Gametocytaemia		14 (9%)	16 (10%)

Data are median (IQR), n (%), or mean (SD), unless otherwise stated. QTcB=Bazett's corrected QT-interval.

* *Pfkelch13* mutations of functional significance, downstream of position 440: *Y493H, R539T*, or *C580Y*.

**Figure 2:**
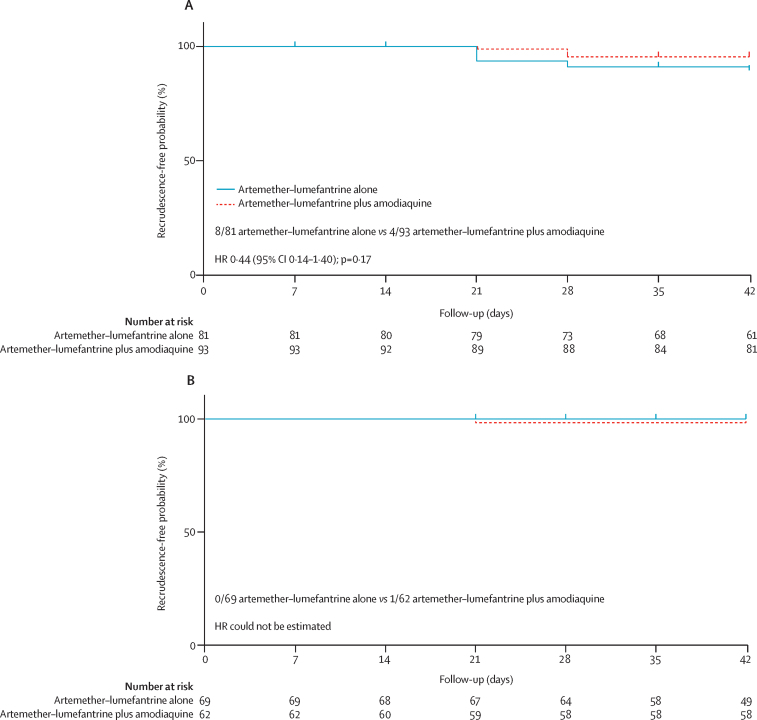
PCR-corrected 42-day treatment efficacy, by artemisinin resistance genotype (A) *pfkelch13* mutated genotype, (B) *pfkelch13* wild-type genotype. Total numbers of PCR-corrected recrudescences in each group are given with the HR and its 95% CI. HR=hazard ratio.

Overall, 42-day treatment efficacy against PCR-confirmed recrudescence was noted in 151 (97%, 95% CI 92–99) of 156 patients with artemether–lumefantrine plus amodiaquine versus 146 (95%, 89–97) of 154 patients with artemether–lumefantrine alone (hazard ratio [HR] for recrudescence 0·6, 95% CI 0·2–1·9, p=0·38). As the total number of recrudescences at each study location was small (table 2), the HRs at each study location are not separately informative, although the overall proportion of patients with PCR-confirmed recrudescent infection appeared somewhat greater in western Cambodia (eight [11%] of 75) than in eastern Cambodia (three [2%] of 184) or Vietnam (two [4%] of 51). Genotyping was possible for 305 (98%) of 310 of the original infections; the remaining five could not be genotyped as they involved low parasite densities, but none of these five infections recurred. Among 174 (57%) of 305 *pfkelch13* mutant infections indicating artemisinin resistance, 42-day efficacy was noted in 89 (96%) of 93 infections treated with artemether–lumefantrine plus amodiaquine versus 73 (90%) of 81 infections with artemether–lumefantrine alone; HR for recrudescence 0·44 (95% CI 0·14–1·40, p=0·17; figure 2). In artemisinin-sensitive infections, there were no recrudescences (of 69 infections) with artemether–lumefantrine alone and one (2%) of 62 with artemether–lumefantrine plus amodiaquine. Recrudescence was more common in infections with mutant *pfkelch13* parasites than with wild-type *pfkelch13* parasites (12 [7%] of 174 *vs* one [<1%] of 131, p=0·009).

**Table 2:** PCR-corrected 42-day treatment efficacy

	Recrudescence-free*by day 42		**Efficacy by day 42 (Kaplan-Meier estimate)**		
	Artemether–lumefantrine alone	Artemether–lumefantrine plus amodiaquine	Artemether–lumefantrine alone	Artemether–lumefantrine plus amodiaquine	Hazard ratio (95% CI)
**Study location**					
Western Cambodia	32/37 (86%)	35/38 (92%)	85% (68–94)	92% (76–97)	0·5 (0·1–2·3)
Eastern Cambodia	91/92 (99%)	90/92 (98%)	99% (91–100)	98% (91–99)	1·9 (0·2–21·5)
Vietnam	23/25 (92%)	26/26 (100%)	92% (72–98)	100% (88–100)	† NA
***Pfkelch* genotype**					
Mutant	73/81 (90%)	89/93 (96%)	90% (80–95)	96% (89–98)	0·4 (0·1–1·4)
Wild-type	69/69 (100%)	61/62 (98%)	100% (96–100)	98% (89–99·8)	† NA
Total	146/154 (95%)	151/156 (97%)	94·5% (89·2–97·2)	96·6% (92·1–98·6)	0·6 (0·2–1·9), p=0·38

Data are n/N (%) or % (95% CI) shown by artemisinin resistance genotype and study location. Hazard ratios, their 95% CI, and p-values computed using Cox's proportional hazards model.

* None were early treatment failures.

† Hazard ratio not estimated as there were no failures in one group.

Of the 13 recrudescent falciparum infections, 12 were detected on days 18–30 (appendix p 2). Although the one other recrudescence was detected on day 45, this patient had not attended follow-up since day 7, so that recrudescence could have become detectable at about the same time as the other 12. There were 33 other recurrences, all but two detected on or after day 28 (25 with artemether–lumefantrine alone *vs* eight with artemether–lumefantrine plus amodiaquine). Four involved a reinfection with a different strain of *P falciparum* and 29 involved *P vivax*. Most of these *P vivax* recurrences were likely relapse of an infection from liver hypnozoite forms of *P vivax*, already present from previous infections, which cannot be detected with current techniques. Mixed infections with falciparum and vivax malaria at baseline were recorded in 49 (16%) of 310 participants, of whom 11 (22%) of 49 had a recurrent infection with *P vivax* during follow-up.

Safety and tolerability were assessed in all patients. Seven serious adverse events were reported, two with artemether–lumefantrine alone and five with artemether–lumefantrine plus amodiaquine. All resolved fully, and on review (appendix p 5) only two were judged as possibly drug related. Of these two patients, one had started artemether–lumefantrine plus amodiaquine but was switched to intravenous artesunate 8 h later because of a blood lactate concentration of 8·6 mmol/L and 5% parasitaemia; as lactate had not been assessed earlier, this WHO criterion for severe malaria might have been present on enrolment. The other had started artemether–lumefantrine plus amodiaquine but was switched to intravenous artesunate 8 h later because of schizont-stage parasites (16/μL) in the blood film, despite not fulfilling WHO criteria for severe malaria. The five judged not to be drug-related involved one blood transfusion (for haemoglobin concentration 6·7 g/dL), one gastritis, one alcohol-related hypertransaminasemia, one dengue co-infection, and one hospitalisation on day 25 for repeated vomiting. There were no deaths in the study.

Apart from these seven serious adverse events, no grade 3–4 symptomatic adverse events were reported in either group (table 3). Vomiting within 1 h of drug administration (hence requiring retreatment) was reported in no patients with artemether–lumefantrine alone versus five of 156 patients treated with artemether–lumefantrine plus amodiaquine. Vomiting at any time during the study period was reported in more patients with artemether–lumefantrine plus amodiaquine than with artemether–lumefantrine alone (table 3).

**Table 3:** Incidence of adverse events

		**All grades**			**Grade 1–2**			**Grade 3–4**		
		Artemether–lumefantrine alone (n=154)	Artemether–lumefantrine plus amodiaquine (n=156)	p value	Artemether–lumefantrine alone (n=154)	Artemether–lumefantrine plus amodiaquine (n=156)	p value	Artemether–lumefantrine alone (n=154)	Artemether–lumefantrine plus amodiaquine (n=156)	p value
Vomiting within 1 h		0	5 (3%)	0·061	..	..	..	..	..	..
Serious adverse event reported*		2 (1%)	5 (3%)	0·45	..	..	..	..	..	..
QTcB >500 ms		0	0	..	..	..	..	..	..	..
QTcB increase >60 ms		0	0	..	..	..	..	..	..	..
Bradycardia (≤54 beats per min)		59 (38%)	95 (61%)	0·0001	..	..	..	..	..	..
Symptoms										
	Vomiting	..	..	..	3 (2%)	12 (8%)	0·031	0	0	..
	Nausea	..	..	..	3 (2%)	11 (7%)	0·05	0	0	..
	Dizziness	..	..	..	9 (6%)	16 (10%)	0·21	0	0	..
	Abdominal pain	..	..	..	19 (12%)	22 (14%)	0·74	0	0	..
	Diarrhoea	..	..	..	13 (8%)	17 (11%)	0·57	0	0	..
	Headache	..	..	..	19 (12%)	20 (13%)	1·00	0	0	..
	Fatigue	..	..	..	11 (7%)	15 (10%)	0·54	0	0	..
	Loss of appetite	..	..	..	9 (6%)	9 (6%)	1·00	0	0	..
	Itching	..	..	..	2 (1%)	5 (3%)	0·45	0	0	..
	Blurred vision	..	..	..	7 (5%)	4 (3%)	0·38	0	0	..
	Sleep disturbance	..	..	..	8 (5%)	12 (8%)	0·49	0	0	..
	Suicidal ideation	..	..	..	0	0	..	0	0	..
	Psychiatric problems	..	..	..	0	0	..	0	0	..
Laboratory abnormality										
	Creatinine	..	..	..	2 (1%)	3 (2%)	1·00	2 (1%)	7 (4%)	0·17
	Total bilirubin	..	..	..	2 (1%)	0	0·25	0	0	..
	Alkaline phosphatase	..	..	..	14 (9%)	8 (5%)	0·19	0	0	..
	Alanyl transferase	..	..	..	16 (10%)	20 (13%)	0·60	0	1 (<1%)	1·00
	Aspartate transferase	..	..	..	15 (10%)	24 (15%)	0·17	2 (1%)	0	0·25
	Anaemia (haemoglobin)	..	..	..	24 (16%)	40 (26%)	0·04	3 (2%)	6 (4%)	0·50
	Leukocytopenia	..	..	..	2 (1%)	0	0·25	0	0	..
	Neutropenia	..	..	..	1 (<1%)	0	0·50	0	0	..
	Thrombocytopenia	..	..	..	37 (24%)	33 (21%)	0·59	1 (<1%)	0	0·50

Data are n (%). Almost all adverse events recorded related to the initial episode of malaria or the 3-day treatment period and preceded discharge, although patients continued to be monitored weekly for 6 weeks. p values were computed using Fisher's exact test. QTcB and bradycardia (defined as ≤54 heartbeats per min) were defined as ever recorded at any of the following timepoints: 4 h, 24 h, 28 h, 48 h, 52 h, 60 h, and 64 h after treatment. QTcB=Bazett's corrected QT-interval.

* These seven serious adverse events reports are detailed in the appendix (p 5). Five were reported as not related to study drug and two cases of severe malaria within 24 h of study entry were reported as possibly related.

No patient in either group ever had an ECG with a prolongation of the Bazett-corrected QT interval (QTcB), or uncorrected QT interval, to more than 500 ms, or an increase in the QTcB of more than 60 ms above baseline. The addition of amodiaquine did, however, cause a small but significant prolongation in the QTcB from baseline to hour 64 (at the time of maximum plasma amodiaquine concentration); the mean increase in QTcB over this period was 5·3 ms (SD 16·0) for artemether–lumefantrine alone versus 11·2 ms (SD 15·4) for artemether–lumefantrine plus amodiaquine, a difference of 6 ms (p=0·0012). Asymptomatic bradycardia (≤54 beats/min) at any time was observed in fewer patients treated with artemether–lumefantrine alone than those treated with artemether–lumefantrine plus amodiaquine (p=0·0001; table 3). Bradycardia was detected mainly at night, when heart rates are slower than during the daytime, with 189 (56%) of 340 episodes recorded between 10 pm and 6 am; the lowest heart rate observed was 42 beats per min.

The frequency of laboratory abnormalities after antimalarial treatment is summarised in table 3. Grade 3–4 abnormalities included transient increases in plasma creatinine (increase by ≥50%, to ≥1·5 × baseline) in nine (3%) of 310 patients with no concentration exceeding 1·3 mg/dL; transient increased aminotransferases (>220 IU/L) in three (1%) of 310; and anaemia (haemoglobin concentration <7 g/dL) in nine (3%) of 310 that did not differ significantly between study groups. Development of mild anaemia (haemoglobin concentration 7·0–10·5 g/dL, grade 1–2) after treatment of the malaria infection occurred in more patients with artemether–lumefantrine plus amodiaquine (p=0·035; table 3).

Parasite clearance half-lives and proportions with parasitaemia persisting to day 3 were similar in the two treatment groups, but varied according to *pfkelch13* genotype (appendix, pp 3–4). Mean parasite clearance half-lives were 6·8 h (SD 2·1) in *pfkelch13*-defined artemisinin-resistant infections, but 3·3 h (1·7) in *pfkelch13* wild-type infections (p<0·0001). Likewise, microscopy-detectable asexual parasites persisting to day 3 occurred in 101 (58%) of 174 *pfkelch13* mutant infections versus 15 (12%) of 130 *pfkelch13* wild-type infections (p<0·0001). At baseline, gametocytaemia was detectable by microscopy in 20 (11%) of 174 *pfkelch13* mutant infections and 10 (8%) of 131 wild-type infections. On day 3 gametocytes remained detectable in nine (45%) of 20 *pfkelch13* mutant infections with gametocytaemia but in none of the ten *pfkelch13* wild-type infections with gametocytaemia.

Pharmacokinetic assessments showed no drug–drug interaction between artemether–lumefantrine and amodiaquine. Plasma concentrations of lumefantrine and its metabolite desbutyl-lumefantrine at day 7 were similar in patients receiving artemether–lumefantrine alone and artemether–lumefantrine plus amodiaquine (appendix p 15). In the subset of 38 Vietnamese patients with frequent blood sampling, the addition of amodiaquine had no material effect on the exposure to artemether–lumefantrine or its metabolites (appendix pp 8–10, 16–22).

**Figure 3:**
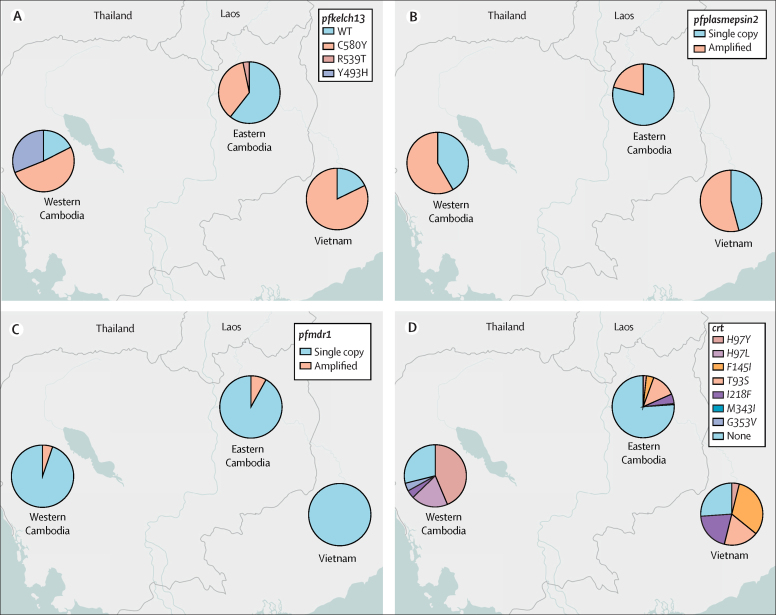
Antimalarial resistance markers in *Plasmodium falciparum* isolates, by study location (A) *Pfkelch13* variants, (B) *pfplasmepsin2* amplification, (C) *pfmdr1* amplification, (D) *Pfcrt* variants.

Overall, 174 (57%) of 305 genotyped infections showed mutations in the propeller region of *pfkelch13*, namely *C580Y* (145 [48%] of 305), *Y493H* (23 [8%] of 305), or *R539T* (six [2%] of 305). Artemisinin resistance defined by the presence of any such mutation was noted in 72 (40%) of 181 infections in eastern Cambodia, 61 (82%) of 74 in western Cambodia, and 41 (82%) of 50 in Vietnam (figure 3). *Pfplasmepsin2* amplification, a marker of piperaquine resistance, was detected in 108 (35%) of 305 genotyped parasites: 43 (58%) of 74 in western Cambodia, 27 (71%) of 38 in Vietnam, and 38 (21%) of 181 in eastern Cambodia. *Pfmdr1* amplification, a marker for mefloquine resistance, was detected in 19 (7%) of 255 infections in Cambodia where artesunate–mefloquine is first-line treatment, but no *pfmdr1* amplification in Vietnam where artesunate–mefloquine is not first-line treatment.

*Pfcrt* mutations, associated with decreased piperaquine sensitivity, were observed in 53 (72%) of 74 genotyped infections in western Cambodia, 37 (74%) of 50 in Vietnam, but only 42 (23%) of 180 in eastern Cambodia, with different *pfcrt* alleles dominating at each location (appendix p 6). *Pfmdr1* and *pfplasmepsin2* amplification were not associated with treatment failure. Of 305 parasite samples, one parasite carried a *pfkelch13* mutation together with amplified *pfmdr1* and *pfplasmepsin2* genes. This infection was detected in a patient in eastern Cambodia and the patient was successfully treated with artemether–lumefantrine plus amodiaquine without recrudescence.

Lumefantrine in-vitro sensitivity assays were restricted to patients with baseline parasitaemia of at least 0·01%. Only 33 (21%) of 154 cryopreserved *P falciparum* samples, all from Cambodian patients, could be cultured successfully. The median IC_50_ was 9 ng/mL (IQR 4–34), but the extremes varied from 0·6 to 77·0 ng/mL, which approaches the conventional cutoff of 80 ng/mL for reduced in-vitro sensitivity to lumefantrine (appendix p 11).

## Discussion

This study confirmed that artemether–lumefantrine plus amodiaquine was safe and showed that the combination was highly effective in curing artemisinin-resistant infections. Only four reinfections with a new *P falciparum* genotype were detected during the 42-day follow-up period, signifying the low level of malaria transmission in the study area.

The chief aim of the study was to assess the effects of adding amodiaquine to artemether–lumefantrine as treatment of uncomplicated falciparum malaria in a population where a substantial proportion of the parasites are artemisinin-resistant, in this study defined by the presence of *pfkelch13* propeller-region mutations. About half the infections were artemisinin resistant, and treatment failure (recrudescence of the original falciparum parasite) was restricted to patients with artemisinin-resistant parasites, except for one recrudescence in an artemisinin-senstitive infection.

The reassuring safety profile of artemether–lumefantrine plus amodiaquine has also been shown in a somewhat larger study^9^ (289 assigned artemether–lumefantrine alone versus 286 artemether–lumefantrine plus amodiaquine), but in that study only 38 patients receiving these drugs had *pfkelch13*-mutant infections, so there were very few recrudesces (three with artemether–lumefantrine alone [one *pfkelch13*-mutant in Laos, two others elsewhere] *vs* none with artemether–lumefantrine plus amodiaquine). Combining the two studies there were in total nine recrudescences in the artemether–lumefantrine group versus four in the artemether–lumefantrine plus amodiaquine group among 212 patients with *pfkelch13*-mutant infections, and two versus one among 673 other patients without *pfkelch13*-mutant infections.

The components of the artemether–lumefantrine plus amodiaquine triple ACT have been on the market for over 25 years and have well described safety and tolerability profiles. Compared with lumefantrine, side-effects such as vomiting, dizziness, and nausea are more frequent with amodiaquine.^9^, ^24^ In the current study, early vomiting within 1 h of drug administration, which jeopardises absorption of the drug, was not observed with artemether–lumefantrine but was observed in 3% of the patients treated with artemether–lumefantrine plus amodiaquine. It is important to consider these gastrointestinal side-effects of amodiaquine, although they affect only a few patients.

This study has reinforced the evidence for cardiac safety of artemether–lumefantrine plus amodiaquine. There was only a small, clinically not relevant prolongation of the QTc interval with the addition of amodiaquine.^9^, ^25^ The increase in mild bradycardia in patients treated with artemether–lumefantrine plus amodiaquine was also observed in the earlier trial of this regimen,^9^ and was not accompanied by clinical symptoms. Additionally, as in the previous trial,^9^ the addition of amodiaquine to artemether–lumefantrine was associated with a temporary increase in plasma creatinine, but this did not affect clinical recovery. The apparent increase in the proportion of patients with anaemia in the present trial, which is mainly caused by the malaria infection itself, might well have been largely or completely a chance finding and is not supported by the previous trial,^9^ in which the numbers developing anaemia were 91 (31%) of 289 with artemether–lumefantrine versus 87 (30%) of 286 artemether–lumefantrine plus amodiaquine. No cases of agranulocytosis or aplastic anaemia were reported in either trial.

The efficacy of artemether–lumefantrine alone against the recrudescence of artemisinin-resistant infections was 90%. Artemether–lumefantrine has not been evaluated recently in either Cambodia and Vietnam, and the current result is reassuring since lower efficacy had been reported previously,^10^, ^11^ with 28-day cure rates of 71% in 2002 and 87% in 2003 in western Cambodia, albeit in the absence of data on lumefantrine plasma concentrations.

Absence of significant lumefantrine resistance is important for the potential future use of artemether–lumefantrine plus amodiaquine in the region. In-vitro lumefantrine sensitivity, expressed as the IC_50_, showed large variation between infecting parasites but did not exceed the cutoff IC_50_ drug concentration for lumefantrine resistance. In Cambodia, reduced efficacy of artesunate-amodiaquine (81%, with 28-day follow-up) was reported in 2016.^26^ There are counteracting resistance mechanisms between lumefantrine and amodiaquine, as shown by the inverse in-vitro sensitivity to the two drugs. In gene-edited *P falciparum* the *N86Y pfmdr1* mutant increases susceptibility to lumefantrine, but increases resistance to amodiaquine.^8^ Additionally, African epidemiological studies report that amodiaquine selects for the *N86*Y and *D1246Y pfmdr1* mutants, whereas lumefantrine selects for the wild type genotype.^7^ However, genetic markers of resistance to amodiaquine are not the same in Africa and Cambodia, and molecular markers for lumefantrine resistance have not been validated for southeast Asia.^26^

The wide deployment of dihydroartemisinin–piperaquine drove a hard genetic sweep through the *P falciparum* parasite populations in Cambodia and southern Vietnam, selecting for a single parasite lineage carrying the *pfkelch* mutation *C580Y* as well as amplification of the *pfplasmepsin2* gene, a marker for piperaquine resistance.^2^ By contrast with the strong predominance of the *C580Y pfkelch* mutation observed previously,^4^, ^9^ there was an increase in the proportion of *Y493H pfkelch13* mutations in western Cambodia, representing a third of all infections, probably related to changes in drug pressure after dihydroartemisinin–piperaquine was abandoned fully in 2017 and replaced by artesunate–mefloquine. In Cambodia, the proportion of parasites carrying multiple copy numbers of *pfmdr1*, a marker of mefloquine resistance, had increased from less than 1% in earlier studies to 7%, probably as a result of artesunate–mefloquine pressure on the parasite population. This dynamic deserves continued surveillance.^5^, ^9^

Pharmacokinetic analyses did not show any effect of the addition of amodiaquine to artemether–lumefantrine on lumefantrine and desbutyl-lumefantrine exposure. The day-7 drug concentrations were not affected, providing evidence that (as per the results from the previous trial^9^) amodiaquine does not interfere with exposure to lumefantrine. Our results indicate that dose adaptations in the artemether–lumefantrine plus amodiaquine triple ACT are not warranted.

This study had some limitations. Most importantly, declines in malaria incidence across the region made recruitment difficult, so during the recruitment period only half the intended number of patients was recruited, of whom only half had artemisinin-resistant infections. Hence, although artemether–lumefantrine appeared to have about 90% efficacy against recrudescence of artemisinin-resistant infections and artemether–lumefantrine plus amodiaquine appeared superior, with about 96% efficacy, this difference was not statistically significant. Second, because this was an open-label trial, assessment of adverse events and their reporting by patients might have been affected by some potential bias. We have no indication for this in the trial data, and the expected increase in gastrointestinal symptoms with the addition of amodiaquine was captured. All microscopy, PCR, and other laboratory assessments were done blind to treatment allocation, so there was no bias in the ascertainment of any of the primary outcomes. Third, patients were censored at the moment of reinfection with another *P falciparum* strain or recurrent infection with *P vivax*, as treatment of this could have obscured the detection of any subsequent recrudescence. However, almost no reinfections happened before the last recrudescent infection, and so this is very unlikely to have materially altered the efficacy estimate. In western Cambodia, some patients working in remote forests had irregular follow-up, but they were eventually assessed and shown to be free of recrudescence. A final limitation is that in-vitro sensitivity to lumefantrine was assessed for only 33 isolates, so low-grade lumefantrine resistance cannot be excluded, and no locally-validated genetic marker of lumefantrine resistance is available.

In the context of increasing artemisinin and ACT partner drug resistance in Cambodia and Vietnam, continued antimalarial drug efficacy is essential for the success of malaria elimination efforts.^1^, ^27^ Artemether–lumefantrine plus amodiaquine provides continued efficacy and is safe and generally well tolerated, except for a slight increase in gastrointestinal adverse events. Importantly, because of the mutual protection against the development of resistance to the two partner drugs, use of triple ACTs could also prolong the useful therapeutic lifetimes of their components, much needed until new antimalarials come to the market.^6^ Artemether–lumefantrine plus amodiaquine could be considered as an alternative first-line treatment in areas with artemisinin-resistant falciparum malaria. More recently, artemisinin resistance has also emerged independently in Africa, in Rwanda and Uganda.^28^, ^29^, ^30^ Although artemether–lumefantrine still shows acceptable efficacy in these countries, the triple ACT artemether–lumefantrine plus amodiaquine could also be an important alternative antimalarial treatment in this region, protecting lumefantrine. To facilitate deployment, a co-packaged formulation of artemether–lumefantrine plus amodiaquine is currently being trialled in large studies in Africa and Asia (NCT03923725 and NCT03939104), and a fixed-dose combination of artemether–lumefantrine plus amodiaquine is being developed.

In conclusion, triple therapy with artemether-lumefantrine plus amodiaquine was an effective and safe treatment for uncomplicated falciparum malaria in a region with multidrug-resistant parasites. The mutual protection by the two partner drugs could prolong the useful therapeutic lifetime of combinations containing lumefantrine, providing an alternative first-line treatment in areas with artemisinin-resistant falciparum malaria in southeast Asia, and elsewhere.

**This online publication has been corrected. The corrected version first appeared at thelancet.com/infection on March 28, 2022**

## Data sharing

Deidentified, individual participant data that underlie this article, along with a data dictionary describing variables in the dataset are available to researchers whose proposed purpose of use is approved by the MORU data access committee. Related documents such as the study protocol and informed consent form are available upon request. To request the dataset, please send a signed data request form to datasharing@tropmedres.ac. The data request form is on the MORU website (https://www.tropmedres.ac/files/moru-bangkok-files/2-dataapplicationformv3-16nov2018.docx). For the purpose of open access, the author has applied a CC-BY public copyright licence to any author-accepted manuscript version arising from this submission.

## Declaration of interests

The Mahidol Oxford Research Unit (MORU) has received funding for other studies of antimalarial treatment from Fosun Pharmaceuticals, which manufactures artemisinin combination therapies. We declare no other competing interests.
