# Correction to *Lancet Infect Dis* 2022; published online March 8. https://doi.org/10.1016/S1473-3099(21)00692-7

**DOI:** 10.1016/S1473-3099(22)00220-1

**Published:** 2022-05

*Peto TJ, Tripura R, Callery JJ, et al. Triple therapy with artemether–lumefantrine plus amodiaquine versus artemether–lumefantrine alone for artemisinin-resistant, uncomplicated falciparum malaria: an open-label, randomised, multicentre trial.* Lancet Infect Dis *2022; published online March 8. https://doi.org/10.1016/S1473-3099(21)00692-7*—In the Research in context panel of this Article, the second sentence of the section on Implications of all the available evidence should have read, “If, as the present study suggests, the addition of amodiaquine to artemether–lumefantrine approximately halves the risk of recrudescence then this could be the preferred treatment in areas with artemisinin resistance.” This correction has been made to the online version as of March 28, 2022, and will be made to the printed version.

