# Supplementary material for: Triple therapy with artemether–lumefantrine plus amodiaquine versus artemether–lumefantrine alone for artemisinin-resistant, uncomplicated falciparum malaria: an open-label, randomised, multicentre trial
Source: Lancet Infect Dis. 2022 Jun;22(6):867–78. doi: 10.1016/S1473-3099(21)00692-7 (PMC9132777; doi:10.1016/S1473-3099(21)00692-7)
Supplement: Supplementary appendix [file mmc1.pdf]

THE LANCET
Infectious Diseases

Supplementary appendix

This appendix formed part of the original submission and has been peer reviewed. We post it as supplied by the authors.

Supplement to: Peto TJ, Tripura R, Callery JJ, et al. Triple therapy with artemether–lumefantrine plus amodiaquine versus artemether–lumefantrine alone for artemisinin-resistant, uncomplicated falciparum malaria: an open-label, randomised, multicentre trial. *Lancet Infect Dis* 2022; published online March 8. https://doi.org/10.1016/S1473-3099(21)00692-7.

Supplementary appendix

Tables

S1. Timing of recrudescent *Pf* infections (PCR-corrected) and of other *Pf* or *Pv* recurrent infections

S2. Parasite and fever clearance time, by *pfkelch13* status and a. study location, b. study treatment

S3. Summary of serious adverse event reports

S4. Antimalarial resistance markers in *P. falciparum* isolates, by study location

S5. Artemether-lumefantrine, amodiaquine, and primaquine dosing schedules

S6. Pharmacokinetic parameter estimates for: a. lumefantrine and desbutyl-lumefantrine, b. artemether and dihydroartemisinin, and c. amodiaquine and desethyl-amodiaquine

Figures

S1. *in vitro* susceptibility to lumefantrine of *P. falciparum* in Cambodia

S2. Kaplan-Meier intent-to-treat analyses of recrudescence-free probability, AL vs AL+AQ, by study location

S3. Kaplan-Meier per-protocol analyses of recrudescence-free probability, AL vs AL+AQ

S4. Effect of amodiaquine on lumefantrine persistence - day 7 plasma concentrations

S5. Individual concentration-time profiles by group (AL vs AL+AQ) for: a. lumefantrine and desbutyl-lumefantrine, b. artemether and dihydroartemisinin, and c. amodiaquine and desethyl-amodiaquine

S6. Pharmacokinetic parameters by group (AL vs AL+AQ) for: a. lumefantrine and desbutyl-lumefantrine, and b. artemether and dihydroartemisinin

S7. Day-7 concentrations of lumefantrine and desbutyl-lumefantrine by group (AL vs AL+AQ)

***Table S1:* Timing of recurrent infections, by species and PCR-correction**

PCR-confirmed weekly numbers with *P. falciparum* recrudescence, and new malarial re-infection without *P. falciparum* recrudescence

	**Recrudescence of *P. falciparum***		**Re-infection by a new *P. falciparum***		**Re-infection by *P. vivax* only****	
**Day* tested**	**AL**	**AL+AQ**	**AL**	**AL+AQ**	**AL**	**AL+AQ**
Day 7	**0**	**0**	**0**	**0**	**0**	**0**
Day 14	**0**	**0**	**0**	**0**	**0**	**0**
Day 21	**5**	**2**[a]	**0**	**0**	**2**	**0**
Day 28	**3**[b]	**3**	**0**	**1**	**6**	**0**
Day 35	**0**	**0**	**3**	**0**	**10**	**1**
Day 42	**0**	**0**	**0**	**0**	**4**	**6**
**Total**	**8**	**5**	**3**	**1**	**22**	**7**

* Rounded to nearest multiple of 7, with tests just after Day 42 rounded to Day 42
** Plasmodium vivax detected by microscopy.
[a] 1 recrudescent infection with artemisinin-sensitive parasite strain (not *pfkelch13* mutant)
[b] Includes 1 participant not seen from day 7 until day 45, when asymptomatically positive

Table S2a: Parasite and fever clearance time, by *Pfkelch13* status and study location

Excludes the 5 patients whose Pf infection was not genotyped

	Pfkelch mutant (N=174)	*Pfkelch* wild-type (N=131)	Mutant minus wild-type, difference (CI)	p-value
**Parasite clearance half-life >5 hours, % (n/N) ***				
Western Cambodia	77 (46/60)	17 (2/12)	60 (36, 84)	0.0002
Eastern Cambodia	85 (60/71)	8 (8/107)	77 (67, 87)	<0.0001
Vietnam	90 (37/41)	22 (2/9)	68 (39, 97)	0.0001
**All 3 study locations**	**83 (143/172)**	**9 (12/128)**	**74 (66, 81)**	**<0.0001**
**Parasite clearance half-life in hours, mean (SD) ***				
Western Cambodia	6.5 (1.9)	4.1 (2.1)	-2.4 (-3.6, -1.2)	<0.0002
Eastern Cambodia	7.1 (2.4)	3.1 (1.6)	-4.0 (-4.6, -3.4)	<0.0001
Vietnam	6.6 (1.6)	4.4 (2.5)	-2.2 (-3.5, -0.9)	0.0016
**All 3 study locations**	**6.8 (2.1)**	**3.3 (1.7)**	**-3.5 (-3.9, -3.0)**	**<0.0001**
**Hours to fever clearance, mean (SD) †**				
Western Cambodia	17.2 (10.5)	15.5 (6.5)	-1.7 (-8.1, 4.7)	0.30
Eastern Cambodia	13.3 (9.4)	14.6 (11.6)	-1.2 (-2.7, 5.2)	0.73
Vietnam	22.9 (13.4)	22.0 (12.0)	+0.9 (-10.9, 9.1)	0.43
**All 3 study locations**	**17.2 (11.5)**	**15.4 (11.2)**	**-1.8 (-4.8, 1.2)**	**0.25**
**Day 3 blood smear still positive, % (n/N) ‡**				
Western Cambodia	57 (35/61)	31 (4/13)	27 (-1, 55)	0.13
Eastern Cambodia	70 (51/72)	8 (9/108)	63 (51, 74)	<0.0001
Vietnam	95 (20/41)	22 (2/9)	73 (44, 100)	0.0001
**All 3 study locations**	**61 (106/174)**	**12 (15/130)**	**49 (40, 59)**	**<0.0001**

p-values computed using Fisher's exact test
* Excludes the 7 patients (including 2 not genotyped) whose initial count was insufficient to estimate the half-life
† Time from baseline to the start of the first 24-hour period <37.5ºC; temperature was recorded every 6 hours
‡ Negative at day 3, or prior clearance or discharge

***Table S2b:* Parasite and fever clearance time, by *Pfkelch13* status and treatment**

	***Pfkelch* mutant (N=174)**	***Pfkelch* wild-type (N=131)**	**Both types (N=305)**
**Parasite clearance half-life >5 hours, % (n/N) ***			
AL	83 (66/80)	10 (7/67)	50 (73/147)
AL+AQ	84 (77/92)	8 (5/61)	54 (82/153)
**Both treatments**	83 (143/172)	9 (12/128)	52 (155/300)
p-value, AL vs AL+AQ	0.84	0.77	0.56
**Parasite clearance half-life in hours, mean (SD) ***			
AL	6.5 (2.1)	3.3 (1.8)	5.0 (2.5)
AL+AQ	7.0 (2.1)	3.3 (1.7)	5.5 (2.6)
**Both treatments**	6.8 (2.1)	3.3 (1.7)	5.3 (2.6)
p-value, AL vs AL+AQ	0.20	0.78	0.12
**Hours to fever clearance, mean (SD) †**			
AL	20 (12.1)	15.7 (12.7)	18.3 (12.6)
AL+AQ	14.5 (10.2)	15.0 (9.3)	14.7 (9.9)
**Both treatments**	17.2 (11.5)	15.4 (11.2)	16.4 (11.4)
p-value, AL vs AL+AQ	0.002	0.75	0.02
**Day 3 blood smear still positive, % (n/N) ‡**			
AL	58 (47/81)	9 (6/68)	36 (53/149)
AL+AQ	63 (59/93)	15 (9/62)	44 (68/155)
**Both treatments**	61 (106/174)	12 (15/130)	40 (121/304)
p-value, AL vs AL+AQ	0.53	0.41	0.16

p-values computed using Fisher's exact test
Excludes the 5 patients with *P. falciparum* infection that was not genotyped
* Excludes the 7 patients (including 2 not genotyped) whose initial count was insufficient to estimate half-life
† Time from baseline to the start of the first 24-hour period <37.5ºC; temperature was recorded every 6 hours
‡ Negative at day 3, or prior clearance or discharge

Table S3: **Summary of Severe Adverse Events**

Number	Study arm	Final diagnosis	Relationship to trial drug	Resolved
1	AL+AQ	(D0, H8) schizonts detected in peripheral blood and high fever prompting switch to IV artesunate	Possible	Yes
2	AL+AQ	Severe malaria, (D0, H8) with hyperlactataemia not assessed on enrolment, rescue treatment with IV artesunate	Possible	Yes
3	AL+AQ	Alcohol-induced grade 4 transaminitis (D7), not symptomatic, hospitalized for observation	Not related	Yes
4	AL+AQ	Hospitalised due to repeated vomiting following ASMQ given for recurrent infection (D25)	Not related	Yes
5	AL+AQ	Gastritis (D11), hospitalized for treatment	Not related	Yes
6	AL	Dengue, with mixed falciparum and vivax co-infection, prolonged hospitalisation with persistent fever (D2)	Not related	Yes
7	AL	Anaemia (Hb=6.7 g/dL on D5), requiring blood transfusion, possible underlying blood disorder (hypochromic and abnormal ‘teardrop’ RBCs)	Not related	Yes

***Table S4:* Proportion with antimalarial resistance markers, by study location**

	Number studied, N	Wild-type *Pfkelch* n (%)	C580Y orY/C *Pfkelch* n (%)	Y493H *Pfkelch* n (%)	R539T *Pfkelch* n (%)	*Plasmepsin* 2/3 amplified n (%)	*Pfmdr1* amplified n (%)	*Pfcrt* mutation n (%)**
Western Cambodia	74	13 (18)	38 (51)	23 (31)	0	43 (58)	4 (5)	53 (72)
Eastern Cambodia	181	109 (60)	66 (36)	0	6 (3)	38 (21)	15 (8)	42 (23)
Vietnam	50	9 (18)	41 (82)	0	0	27 (54)	0	37 (74)
**All three study locations**	**305***	**131 (43)**	**145 (48)**	**23 (8)**	**6 (2)**	**108 (35)**	**19 (6)**	**132 (43)**

* 5 not genotyped: Vietnam 1, Western Cambodia 1, Eastern Cambodia 3
** *crt* mutation T93, H97, F145, I218, M343, or G353

Table S5: **Artemether-lumefantrine, amodiaquine, and primaquine dosing schedules**

**AL dosing schedule: No. of tablets recommended at approximate timing of dosing. One tablet contains 20mg artemether and 120mg lumefantrine**						
**Weight: Kg**	**0 h**	**8 h**	**24 h**	**36 h**	**48 h**	**60 h**
**5-14.9**	**1**	**1**	**1**	**1**	**1**	**1**
**15-24.9**	**2**	**2**	**2**	**2**	**2**	**2**
**25-34.9**	**3**	**3**	**3**	**3**	**3**	**3**
**≥35**	**4**	**4**	**4**	**4**	**4**	**4**

**Amodiaquine dosing schedule: No. of tablets recommended at approximate timing of dosing. One tablet contains 150mg Amodiaquine**						
**Weight: Kg**	**0 h**	**8 h**	**24 h**	**36 h**	**48 h**	**60 h**
**5-14.9**	**0.5**	**0**	**0.5**	**0**	**0.5**	**0**
**15-24.9**	**0.5**	**0.5**	**0.5**	**0.5**	**0.5**	**0.5**
**25-34.9**	**1**	**1**	**1**	**1**	**1**	**1**
**≥35**	**1.5**	**1.5**	**1.5**	**1.5**	**1.5**	**1.5**

**Primaquine dosing schedule: No. of tablets recommended on day 1 only. One tablet contains 7.5mg primaquine**	
**Weight: Kg**	**No. of tablets recommended (once only)**
**<25**	**0.5 tablet on day 1**
**25-50**	**1 tablet on day 1**
**>50**	**2 tablets on day 1**

Table S6a: **Lumefantrine and desbutyl-lumefantrine PK parameters**

**Pharmacokinetic parameter**	**Lumefantrine PK, median (IQR)**		**Desbutyl-lumefantrine PK, median (IQR)**	
	**AL alone (n = 20)**	**AL+AQ (n = 18)**	**AL alone (n = 20)**	**AL+AQ (n = 18)**
C_{MAX} after first dose (ng/mL)	5900 (4320-7750)	5130 (3940-7790)	4.46 (3.38-7.16)	4.13 (2.48-7.04)
C_{MAX} /Dose after first dose (ng/mL) /(mg/kg)	172 (133-227)	138 (123-254)	0.135 (0.089-0.209)	0.112 (0.075-0.179)
C_{MAX} after last dose (ng/mL)	7230 (4420-10 700)	8810 (6180-10 700)	24.3 (18.3-50.7)	38.3 (27.9-44.8)
C_{MAX} /Dose after last dose (ng/mL) /(mg/kg)	226 (129-313)	254 (181-375)	0.663 (0.528-1.33)	0.945 (0.707-1.27)
T_{MAX} after first dose (hours)	7.83 (6.00-7.88)	7.88 (5.98-7.95)	7.89 (7.83-7.92)	7.90 (7.84-7.92)
$AUC_{0\text{-}8\ hr}$ (hours × ng/mL)	23,300 (17,600-25,500)	20,800 (15,500-30,300)	15.0 (9.31-17.8)	11.5 (9.12-20.4)
$AUC_{0\text{-}8\ hr}$ /Dose (hours × ng/mL) /(mg/kg)	674 (512-761)	596 (476-1,024)	0.411 (0.254-0.499)	0.324 (0.255-0.594)
$AUC_{60\text{-}LAST}$ (hours × µg/mL)	340 (216-483)	406 (292-550)	3.70 (2.90-6.28)	4.82 (3.68-6.02)
$AUC_{60\text{-}LAST}$ /Dose (hours × µg/mL) /(mg/kg)	10.9 (6.4-14.9)	13.6 (8.6-18.1)	0.103 (0.080-0.165)	0.138 (0.087-0.166)
AUC_{LAST} (hours × µg/mL)	348 (216-491)	397 (300-550)	3.73 (2.78-6.24)	4.86 (3.26-6.10)
AUC_{LAST} /Dose (hours × µg/mL) /(mg/kg)	11.2 (6.3-15.0)	11.9 (8.6-18.4)	0.103 (0.072-0.163)	0.134 (0.086-0.167)
$t_{1/2}$ (days)	8.96 (7.74-9.99)	9.61 (7.34-11.3)	10.4 (8.3-11.6)	9.25 (7.92-11.1)

***Table S6b:* Artemether and dihydroartemisinin median (IQR) PK parameters**

**Pharmacokinetic parameter**	**Artemether, median (IQR)**		**Dihydroartemisinin, median (IQR)**	
	**AL alone (n = 20)**	**AL+AQ (n = 18)**	**AL alone (n = 20)**	**AL+AQ (n = 18)**
C_{MAX} (ng/mL)	270 (141-348)	239 (174-325)	97.2 (75.0-134)	76.1 (51.9-105)
C_{MAX} /Dose (ng/mL) /(mg/kg)	15.1 (7.8-19.3)	16.1 (9.4-20.7)	6.27 (3.66-7.42)	4.27 (2.60-5.84)
T_{MAX} (hours)	2.00 (1.76-3.96)	2.00 (1.30-3.99)	2.00 (2.00-3.96)	2.00 (1.98-4.00)
$AUC_{0\text{-}8\ hr}$ (hours × ng/mL)	657 (517-902)	640 (549-863)	288 (235-378)	248 (157-319)
$AUC_{0\text{-}8\ hr}$ /Dose (hours × ng/mL) /(mg/kg)	30.0 (28.9-61.2)	43.2 (40.2-57.0)	16.8 (13.6-20.2)	13.8 (9.37-17.5)
$t_{1/2}$ (hours)	1.85 (1.61-2.31)	1.90 (1.35-2.35)	1.18 (1.01-1.34)	1.22 (1.06-1.68)

Table S6c: **Amodiaquine and desethyl-amodiaquine median (IQR) PK parameters**

**Pharmacokinetic parameter**	**Amodiaquine, median (IQR), for AL+AQ (n = 18)**	**Desethyl-amodiaquine, median (IQR), for AL+AQ (n = 18)**
C_{MAX} (ng/mL)	8.93 (6.53-11.3)	203 (156-251)
C_{MAX} /Dose (ng/mL) /(mg/kg)	295 (211-335)	5,610 (4,970-6,430)
T_{MAX} (hours)	2.0 (1.01-2.0)	64 (64-64)
AUC_{LAST} (hours × ng/mL)	34.8 (30.8-52.3)	23,800 (20,400-27,300)
AUC_{LAST}/Dose (hours × µg/mL) /(mg/kg)	1.22 (0.933-1.54)	681 (554-816)
$t_{1/2}$ (hours)	5.06 (3.19-7.93)	227 (182-295)

***Figure S1: In vitro* susceptibility of Cambodian *P. falciparum* clinical isolates to lumefantrine (N=33)**

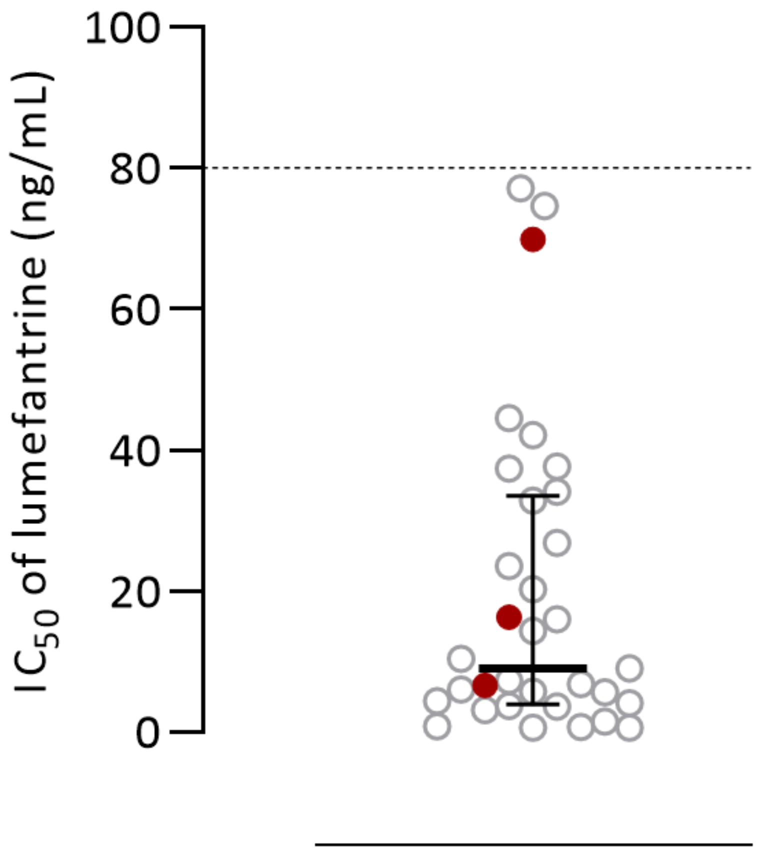

Median IC_{50} of *P. falciparum* asexual parasites isolated in Cambodia during TACT-CV after 48 h exposure with lumefantrine in the schizont maturation assay. Error bars denote IQR, grey circles denote IC_{50} of individual isolates, dotted line denotes *in vitro* lumefantrine resistance cut-off. Red coloured dots indicate recrudescent infections (treatment failures). IC_{50} of lumefantrine (ng/mL) in Eastern Cambodia (N=19), median 14.3 (IQR 4.1-32.8, range 0.7- 74.6); Western Cambodia (N=14), median 6.9 (IQR 3.6-36.1, range 0.6-77.2).

Figure S2a: **Kaplan-Meier plot comparing recrudescence-free probability between the AL and AL+AQ arms**

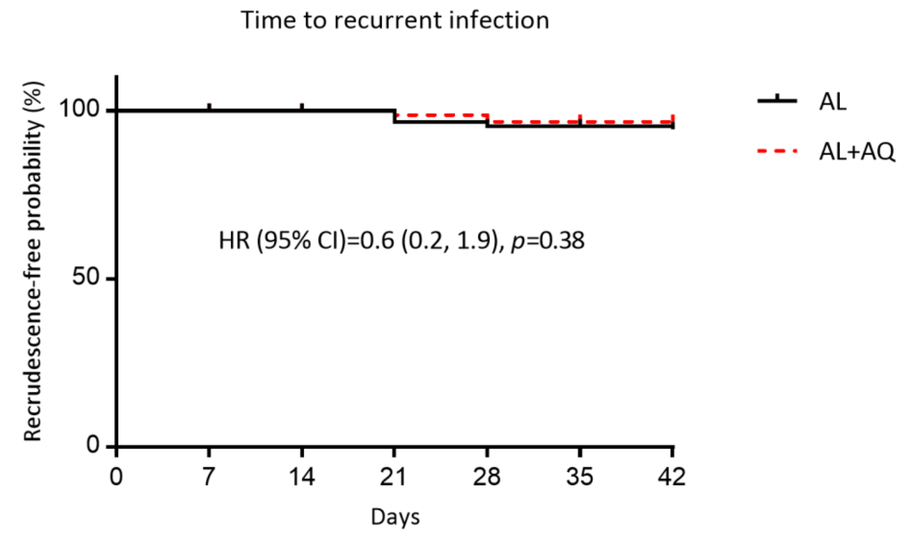

Number at risk

AL:	154	154	152	150	141	130	114
Al+AQ:	156	156	153	149	147	143	140

Figure S2b: **Kaplan-Meier plot comparing the recrudescence-free probability between the AL and AL+AQ arms in western Cambodia**

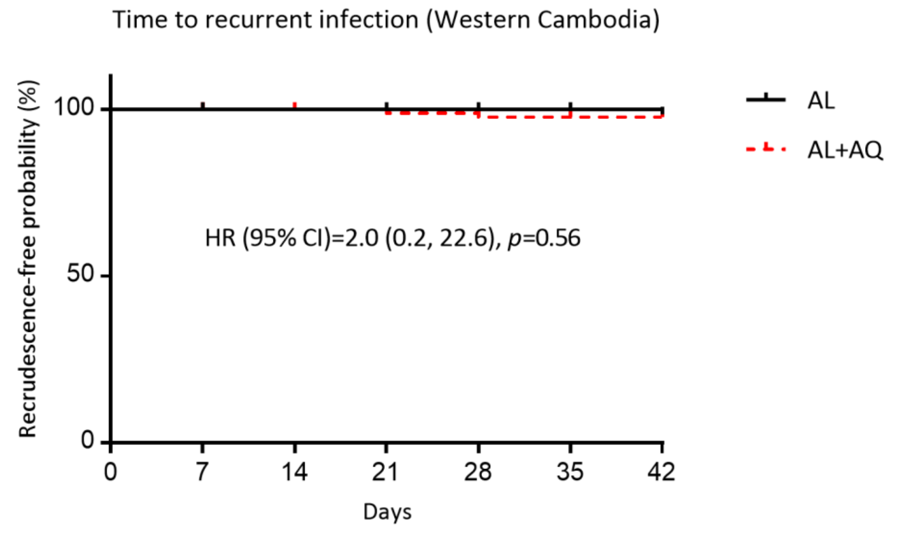

Number at risk

AL:	92	92	91	91	88	82	72
Al+AQ:	92	92	89	87	86	85	84

Figure S2c: **Kaplan-Meier plot comparing the recrudescence-free probability between the AL and AL+AQ arms in eastern Cambodia**

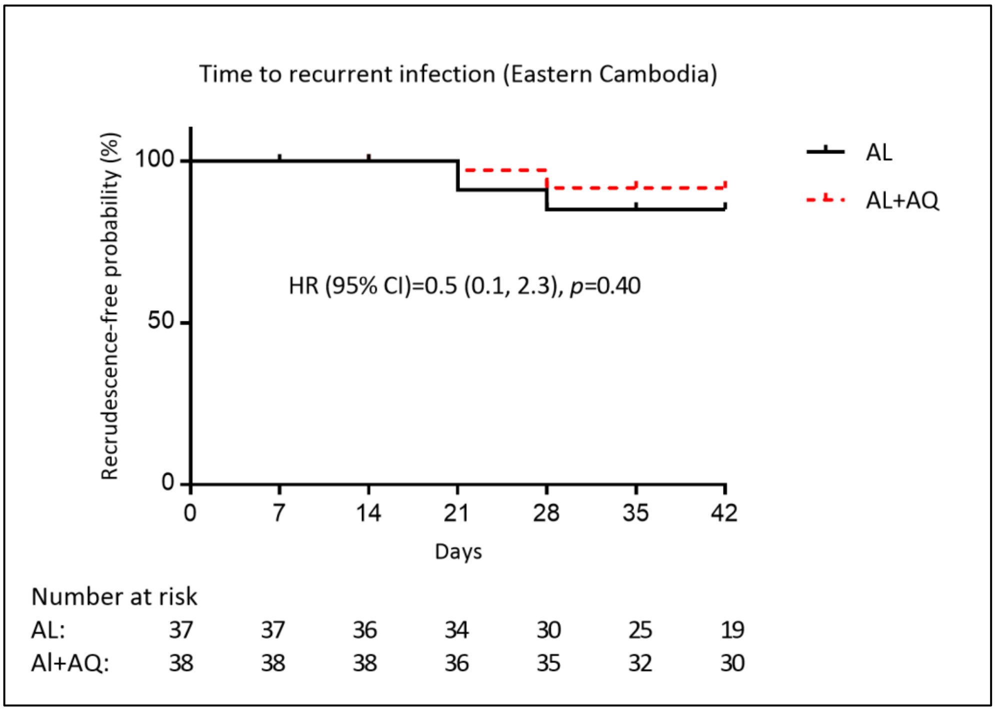

Figure S2d: **Kaplan-Meier plot comparing the recrudescence-free probability between the AL and AL+AQ arms in southern Vietnam**

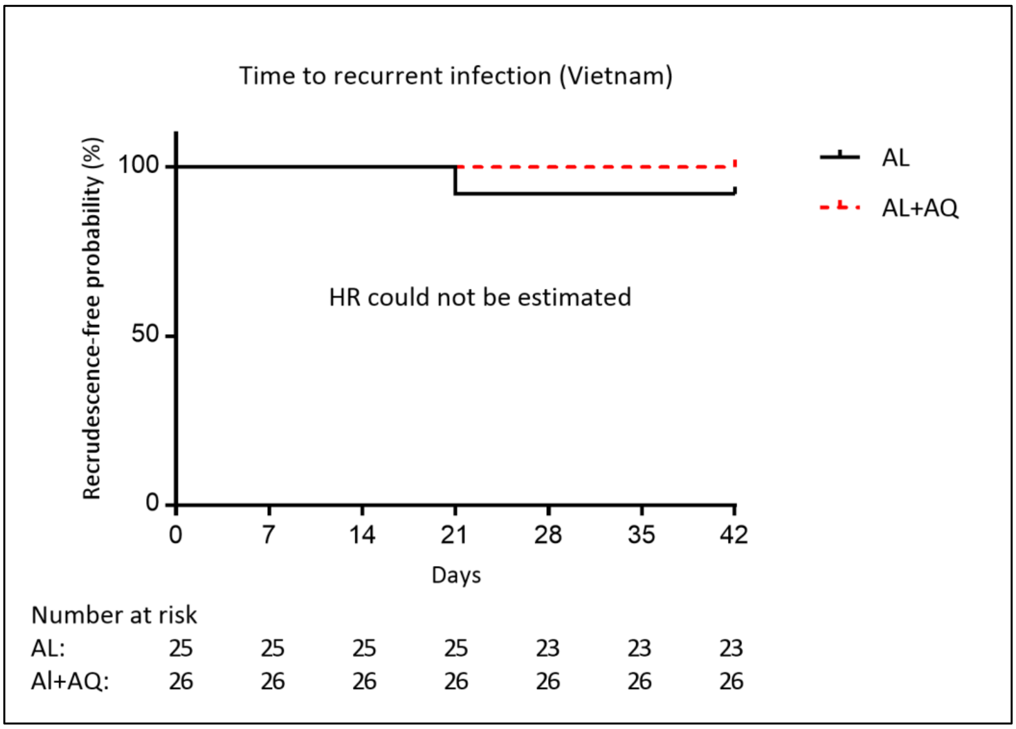

Figure S3: **Kaplan-Meier plot comparing the recrudescence-free probability between the AL and AL+AQ arms, per protocol**

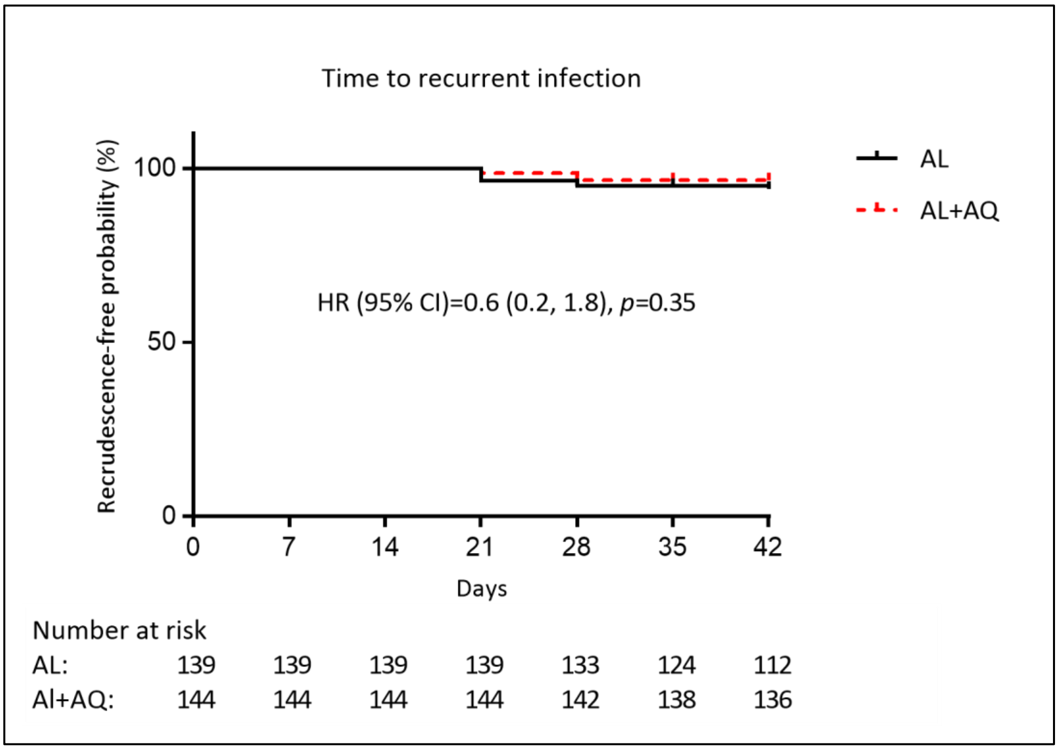

Figure S3: **Effect of amodiaquine on lumefantrine persistence - day 7 plasma concentrations**

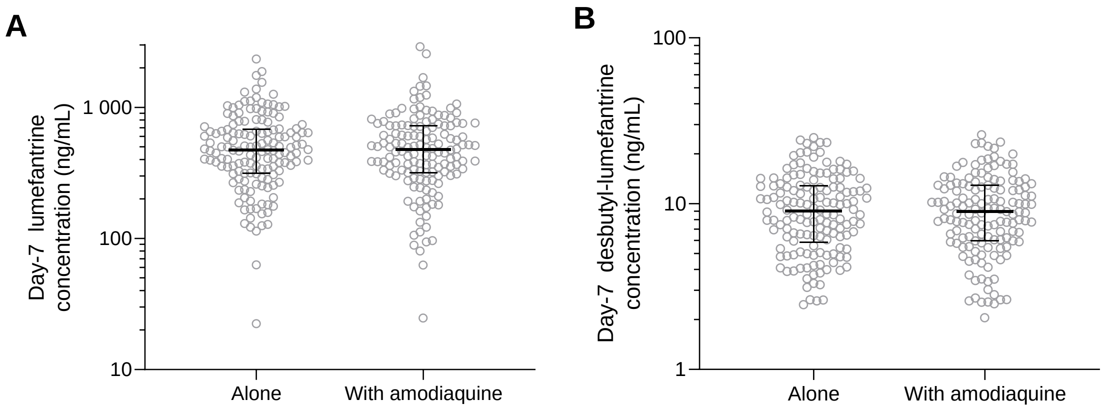

Lumefantrine (A) and desbutyl-lumefantrine (B), AL alone versus AL+AQ. Day-7 concentrations (ng/mL) were summarised from all patients (148 AL alone, 145 AL+AQ). Lumefantrine, AL alone median 474 (IQR 316-680); AL+AQ median 478 (IQR 318-725). Desbutyl-lumefantrine, AL alone median 9 (IQR 6-13); AL+AQ median 9 (IQR 6-13).

Figure S5a: **Individual lumefantrine and desbutyl-lumefantrine concentration-time profiles, by treatment arm**

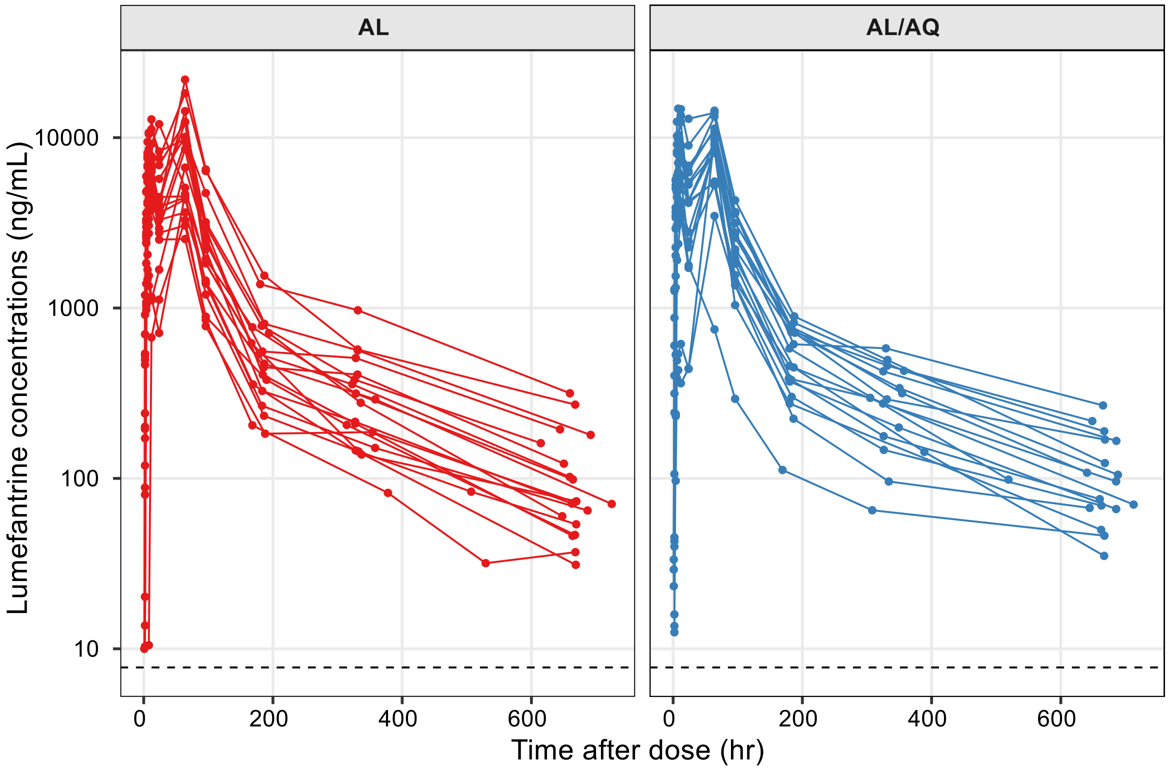

The horizontal dashed line represents the lower quantification limit of 7.8 ng/mL.

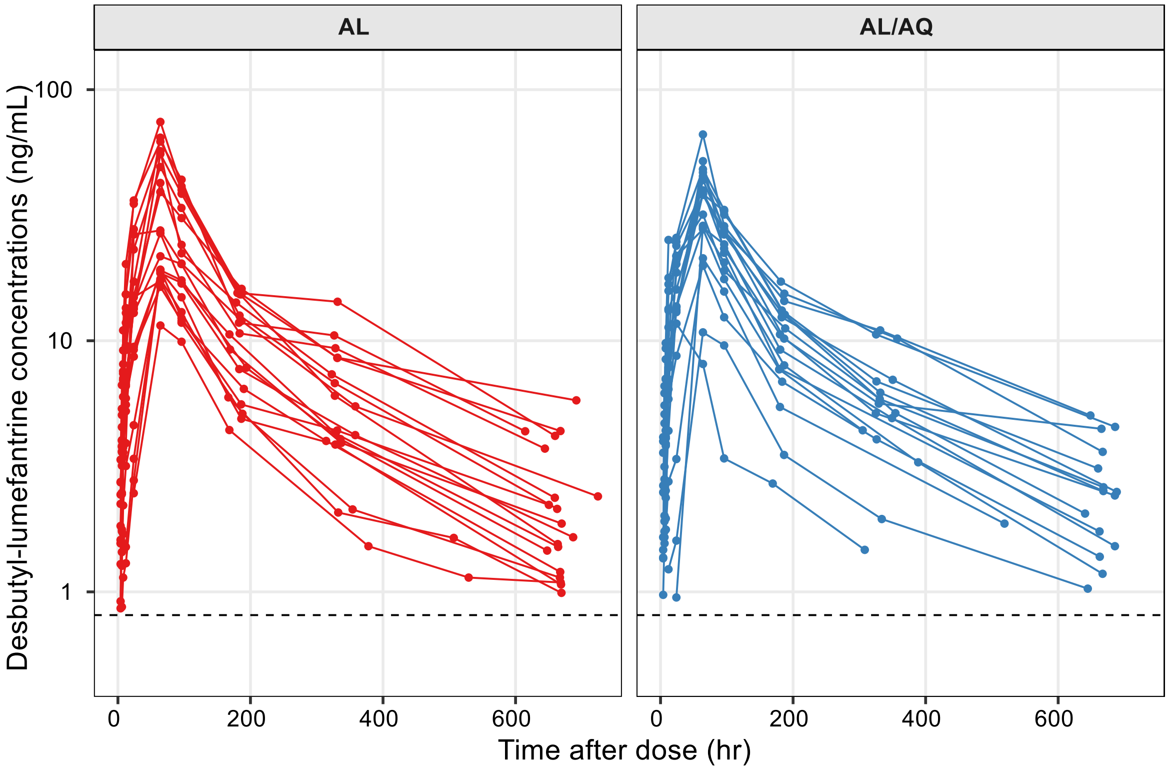

The horizontal dashed line represents the lower quantification limit of 0.81 ng/mL.

Figure S5b: **Individual artemether and dihydroartemisinin concentration-time profiles, by treatment arm**

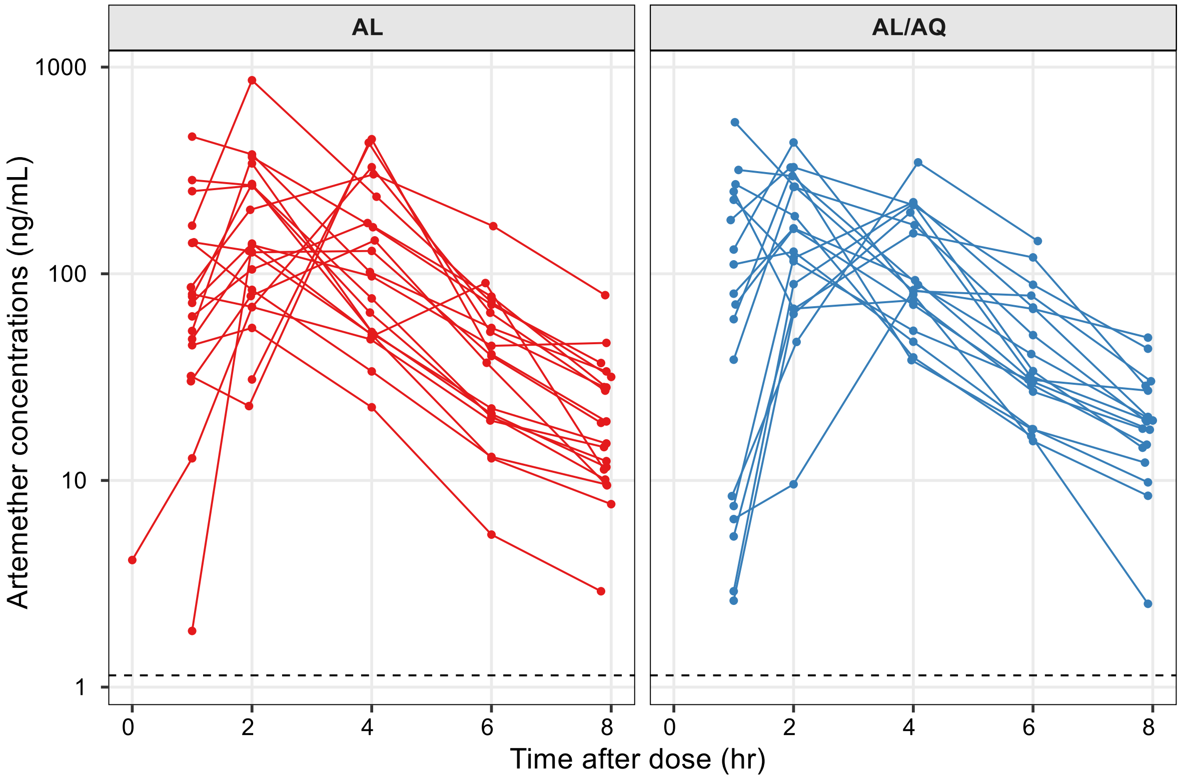

The horizontal dashed line represents the bioanalytical measurement limit of 1.14 ng/mL.

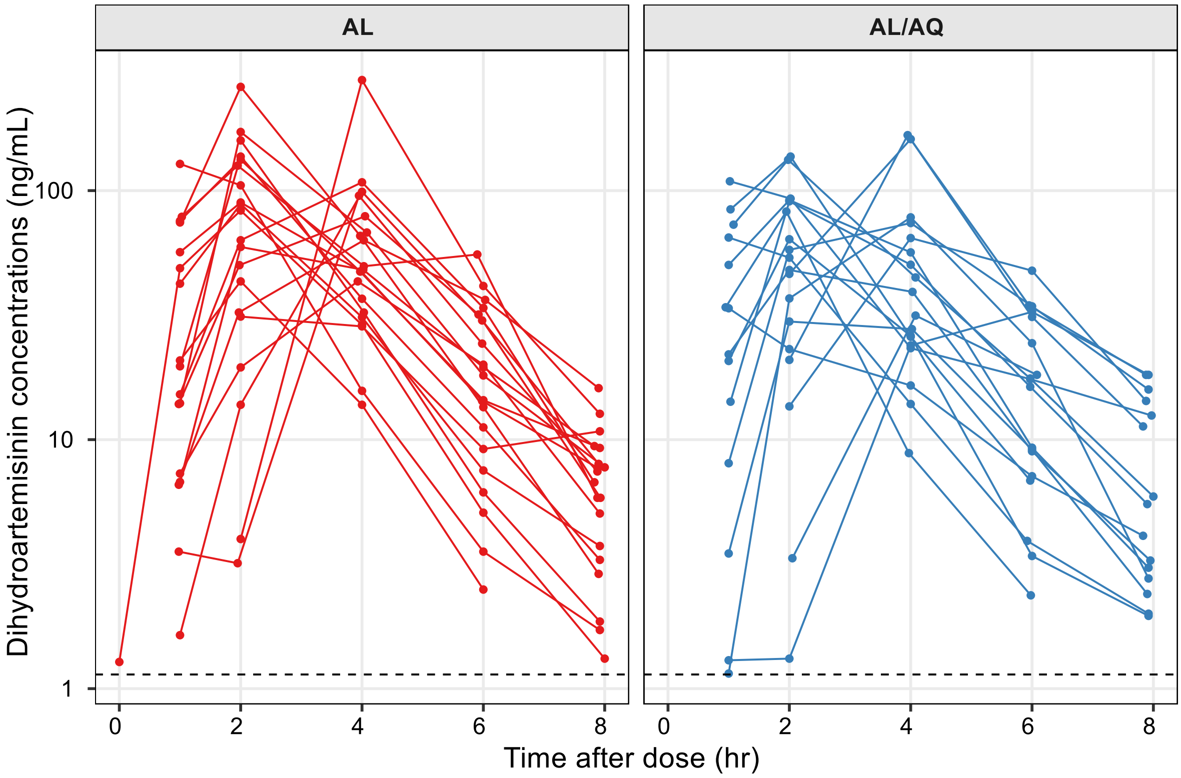

The horizontal dashed line represents the bioanalytical measurement limit of 1.14 ng/mL.

Figure S5c: **Individual concentration-time profiles among 18 patients allocated AL+AQ for (A) amodiaquine, and (B) desethyl-amodiaquine**

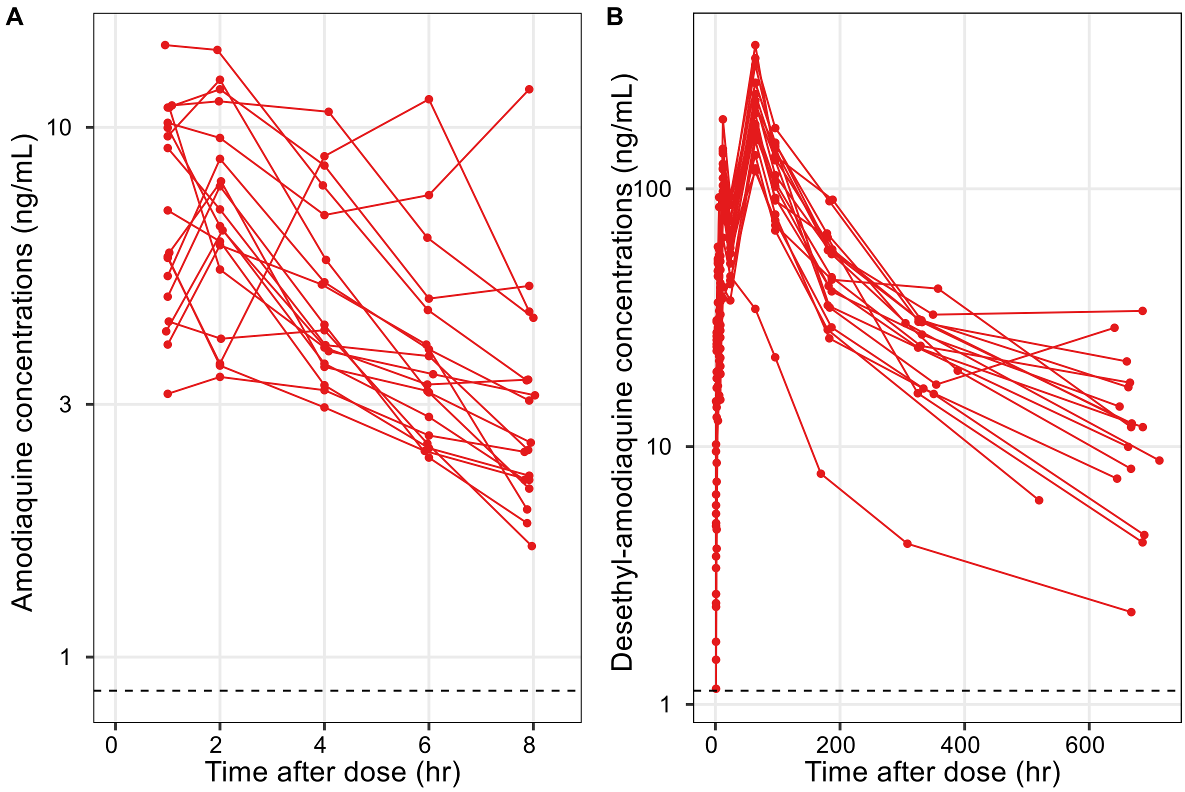

The horizontal dashed lines represent the bioanalytical measurement limits of 0.864 and 1.13 ng/mL.

***Figure S6a:* Comparison of PK parameters for lumefantrine between patients receiving AL alone and AL+AQ**

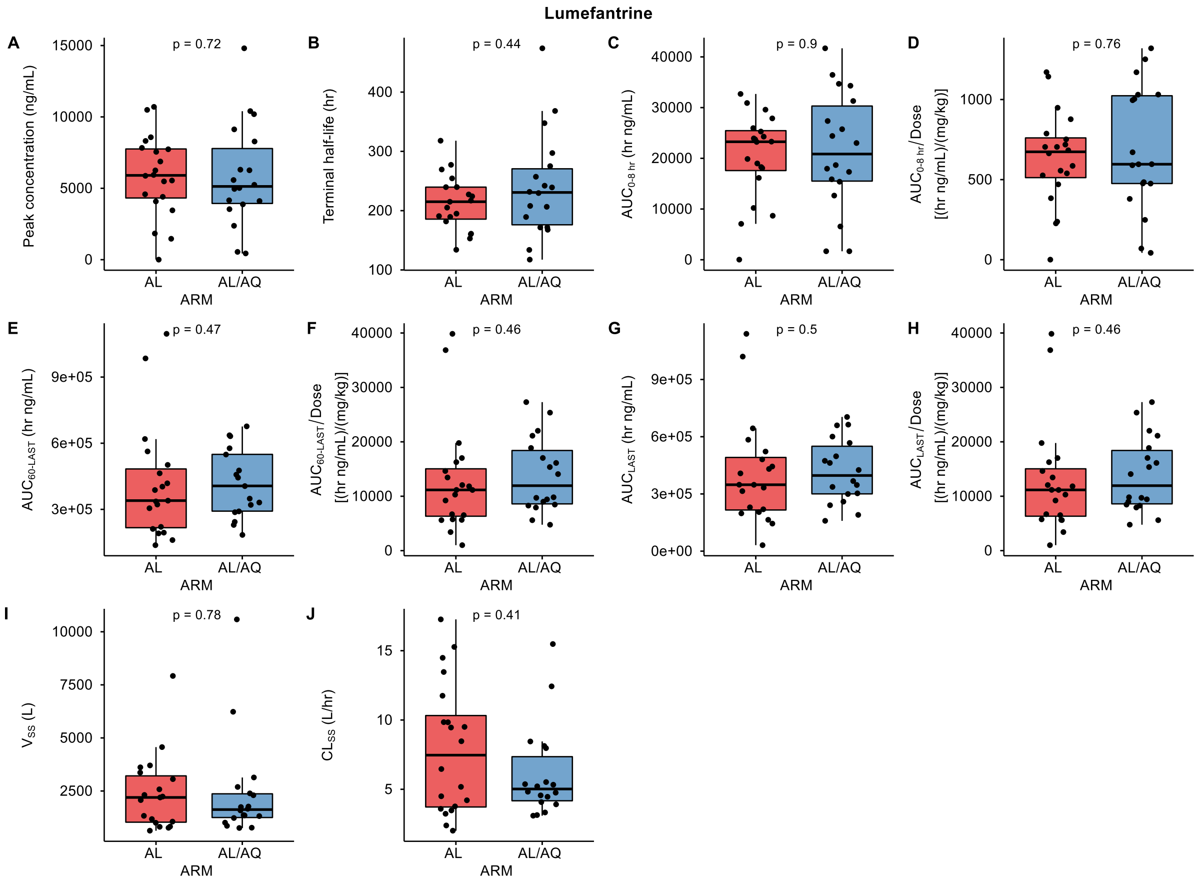

(A) peak concentration after the first dose, (B) terminal half-life, (C) area under the concentration-time curve after first dose ($AUC_{0\text{-}8\ hr}$), (D) dose-normalised $AUC_{0\text{-}8\ hr}$, (E) area under the concentration-time curve after the last dose ($AUC_{60\text{-}LAST}$), (F) dose-normalised $AUC_{60\text{-}LAST}$ (G) overall area under the concentration-time curve (AUC_{LAST}), (H) dose-normalised AUC_{LAST} (I) oral volume of distribution, and (J) oral clearance. P-values were calculated using the non-parametric Mann-Whitney U-test.

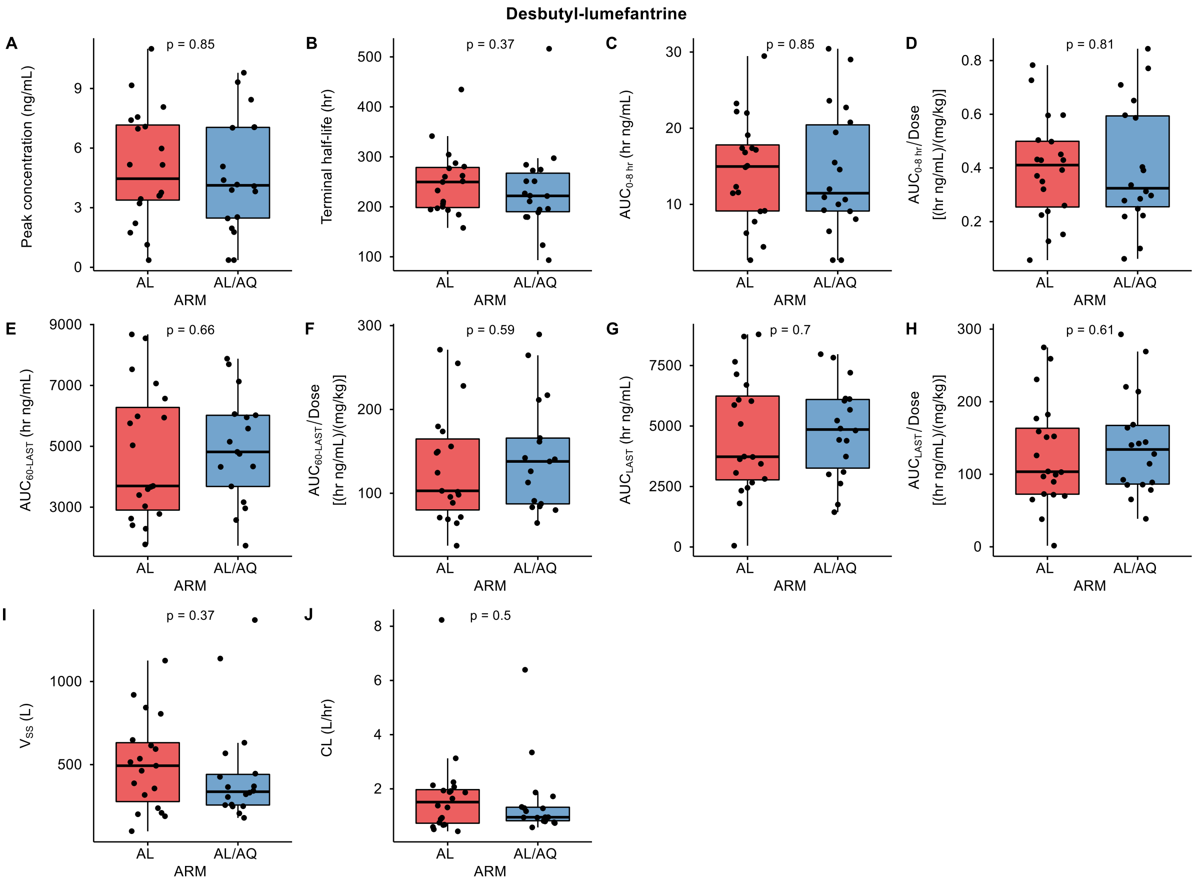

(A) peak concentration after the first dose, (B) terminal half-life, (C) area under the concentration-time curve after first dose ($AUC_{0\text{-}8\ hr}$), (D) dose-normalised $AUC_{0\text{-}8\ hr}$, (E) area under the concentration-time curve after the last dose ($AUC_{60\text{-}LAST}$), (F) dose-normalised $AUC_{60\text{-}LAST}$ (G) overall area under the concentration-time curve (AUC_{LAST}), (H) dose-normalised AUC_{LAST} (I) oral volume of distribution, and (J) oral clearance. P-values were calculated using the non-parametric Mann-Whitney U-test.

Figure S6b: **PK parameters for artemether and dihydroartemisinin – comparisons between patients receiving AL alone and AL+AQ**

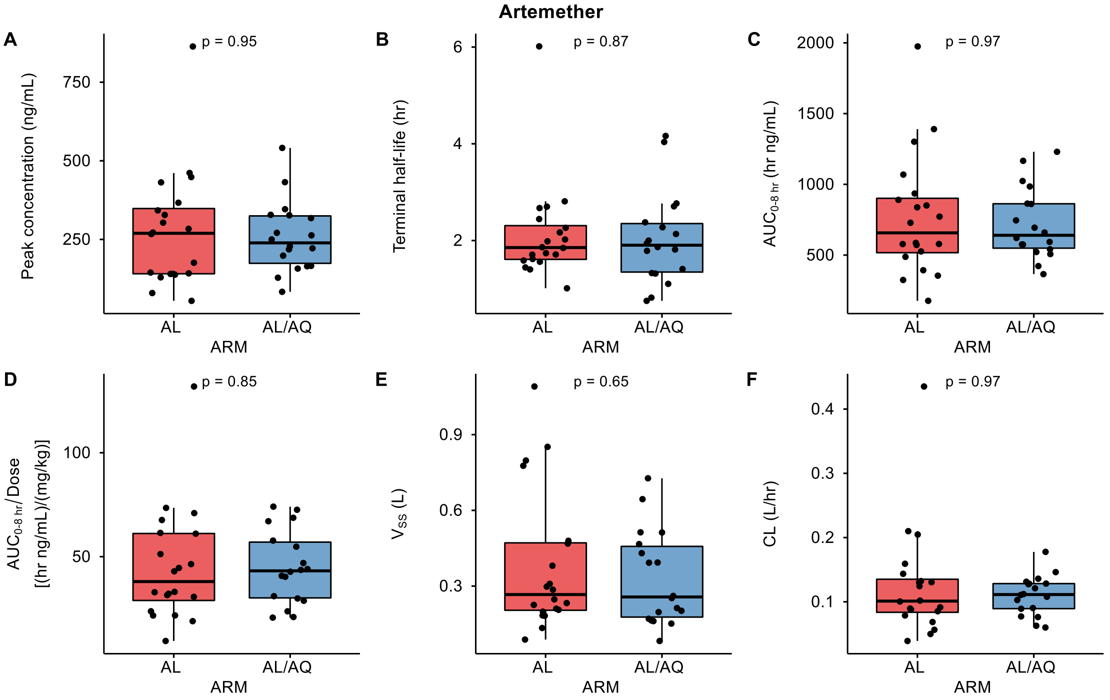

(A) peak concentration, (B) terminal half-life, (C) area under the concentration-time curve after the first dose ($AUC_{0\text{-}8\ hr}$), (D) dose-normalised $AUC_{0\text{-}8\ hr}$, (E) oral volume of distribution, and (F) oral clearance. P-values were calculated using the non-parametric Mann-Whitney U-test.

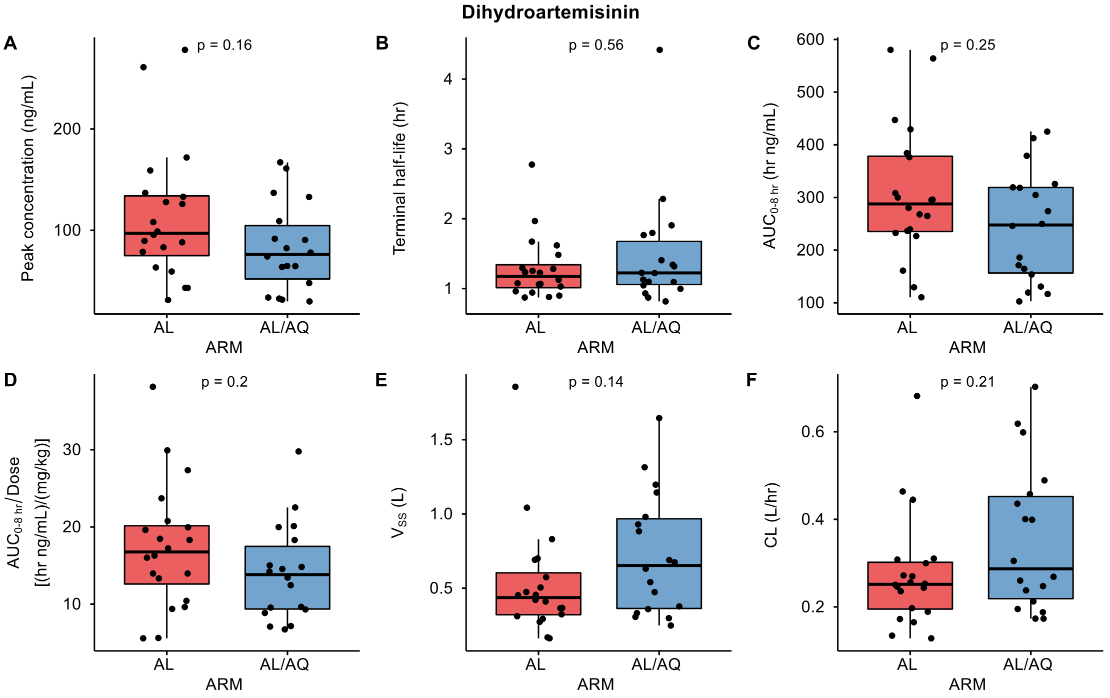

(A) peak concentration, (B) terminal half-life, (C) area under the concentration-time curve after the first dose ($AUC_{0\text{-}8\ hr}$), (D) dose-normalised $AUC_{0\text{-}8\ hr}$, (E) oral volume of distribution, and (F) oral clearance. P-values were calculated using the non-parametric Mann-Whitney U-test.

Figure S7: **Day-7 concentrations of lumefantrine and desbutyl-lumefantrine by group (AL vs AL+AQ)**

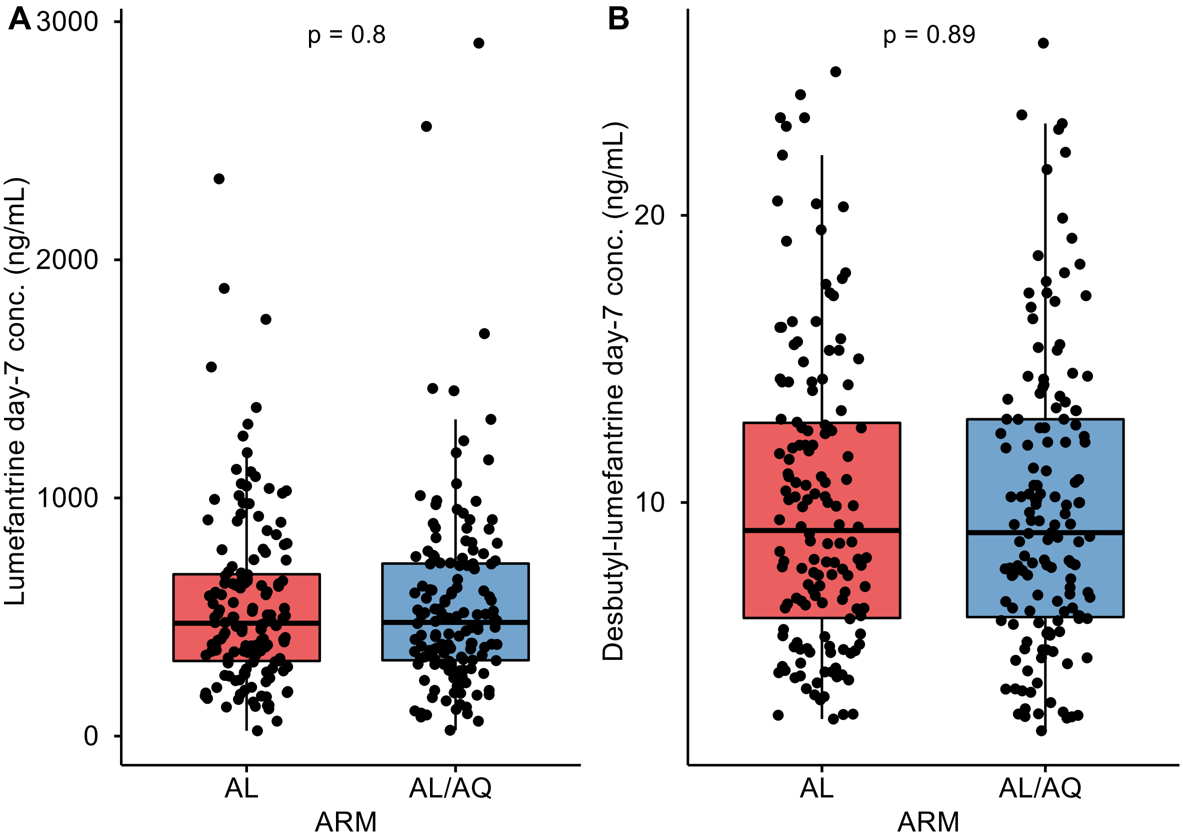

P-values were calculated using the non-parametric Mann-Whitney U-test.
